# Multi-omics signatures in new-onset diabetes predict metabolic response to dietary inulin: findings from an observational study followed by an interventional trial

**DOI:** 10.1038/s41387-023-00235-5

**Published:** 2023-04-21

**Authors:** N. Ďásková, I. Modos, M. Krbcová, M. Kuzma, H. Pelantová, J. Hradecký, M. Heczková, M. Bratová, P. Videňská, P. Šplíchalová, M. Králová, M. Heniková, J. Potočková, A. Ouřadová, R. Landberg, T. Kühn, M. Cahová, J. Gojda

**Affiliations:** 1 First Faculty of Medicine, Charles University, Prague, Czech Republic; 2 Institute for Clinical and Experimental Medicine, Prague, Czech Republic; 3 Department of Internal Medicine, Kralovske Vinohrady University Hospital and Third Faculty of Medicine, Charles University, Prague, Czech Republic; 4 Institute of Microbiology of the CAS, Prague, Czech Republic; 5 Faculty of Forestry and Wood Sciences, Czech University of Life Sciences, Prague, Czech Republic; 6 Mendel University, Department of Chemistry and Biochemistry, Brno, Czech Republic; 7 RECETOX, Faculty of Science Masaryk University, Brno, Czech Republic; 8 Ambis University, Department of Economics and Management, Prague, Czech Republic; 9 Division of Food and Nutrition Science, Department of Biology and Biological Engineering, Chalmers University of Technology, Goteborg, Sweden; 10 Institute of Global Food Security, Queen’s University Belfast, Belfast, UK; 11 Heidelberg Institute of Global Health (HIGH), Medical Faculty and University Hospital, Heidelberg University, Heidelberg, Germany; 12 Present Address: Institute for Clinical and Experimental Medicine, Prague, Czech Republic

**Keywords:** Translational research, Biostatistics

## Abstract

**Aim:**

The metabolic performance of the gut microbiota contributes to the onset of type 2 diabetes. However, targeted dietary interventions are limited by the highly variable inter-individual response. We hypothesized (1) that the composition of the complex gut microbiome and metabolome (MIME) differ across metabolic spectra (lean-obese-diabetes); (2) that specific MIME patterns could explain the differential responses to dietary inulin; and (3) that the response can be predicted based on baseline MIME signature and clinical characteristics.

**Method:**

Forty-nine patients with newly diagnosed pre/diabetes (DM), 66 metabolically healthy overweight/obese (OB), and 32 healthy lean (LH) volunteers were compared in a cross-sectional case-control study integrating clinical variables, dietary intake, gut microbiome, and fecal/serum metabolomes (16 S rRNA sequencing, metabolomics profiling). Subsequently, 27 DM were recruited for a predictive study: 3 months of dietary inulin (10 g/day) intervention.

**Results:**

MIME composition was different between groups. While the DM and LH groups represented opposite poles of the abundance spectrum, OB was closer to DM. Inulin supplementation was associated with an overall improvement in glycemic indices, though the response was very variable, with a shift in microbiome composition toward a more favorable profile and increased serum butyric and propionic acid concentrations. The improved glycemic outcomes of inulin treatment were dependent on better baseline glycemic status and variables related to the gut microbiota, including the abundance of certain bacterial taxa (i.e., *Blautia, Eubacterium halii group, Lachnoclostridium, Ruminiclostridium, Dialister*, or *Phascolarctobacterium*), serum concentrations of branched-chain amino acid derivatives and asparagine, and fecal concentrations of indole and several other volatile organic compounds.

**Conclusion:**

We demonstrated that obesity is a stronger determinant of different MIME patterns than impaired glucose metabolism. The large inter-individual variability in the metabolic effects of dietary inulin was explained by differences in baseline glycemic status and MIME signatures. These could be further validated to personalize nutritional interventions in patients with newly diagnosed diabetes.

Obesity and its associated metabolic diseases, including type 2 diabetes, currently represent one of the greatest challenges to global health care [1]. Recently, it has been suggested that the composition and performance of the gut microbiota contribute to individual risks. The critical role of the gut microbiota in the development of obesity was suggested by a seminal study by Turnbaugh [2], followed by others confirming differences in microbiota composition between lean and obese individuals [3, 4]. Further research showed an association between the gut microbiota and the development of type 2 diabetes [5–8], with evidence of a specific gut microbiota signature characteristic of prediabetes [9, 10]. However, while many studies suggest that type 2 diabetes is associated with gut dysbiosis [11], results on the composition and function of the microbiota are inconsistent and sometimes contradictory. For example, α-diversity has been reported to be significantly lower [6, 12, 13], not significantly reduced [14], or comparable to nondiabetic subjects in patients with T2D [15, 16]. Most studies report significant differences in the composition of the gut microbiota between diseased and healthy subjects [17], but they differ greatly with respect to specific taxa. Some studies show that T2D is associated with an increased Firmicutes/Bacteroidetes ratio [6, 13, 14, 18, 19], whereas others report a significant increase [14, 18] or decrease [6, 13] in Proteobacteria. At the genus level, there are few dysregulated taxa that have been consistently reported, i.e., an increase in *Streptococcus* [9, 15, 20], *Escherichia* [15, 21, 22], *Veillonella* [6, 21], Lactobacillus [13, 18, 23], and *Collinsella* [12, 15]; decrease in *Akkermansia* [15, 18], *Dialister* [15, 19], *Haemophilus* [12, 15], *Roseburia* [12, 15], and *Faecalibacterium* [10, 12, 13], whereas many others show changes in both directions [17]. Diet composition is a known risk factor for the development of type 2 diabetes. In addition to direct effects on host physiology, diet plays an important role in shaping the microbiome, thereby influencing its metabolic program [24]. Therefore, dietary interventions focused on modulating the composition and/or performance of the gut microbiota appear to be a promising therapeutic target. Supplementation with prebiotic supplements, and dietary fiber in particular, is often recommended as a beneficial treatment for non-communicable diseases, but controlled clinical trials indicate pronounced differences in response to treatment, with considerable personal variability [25]. The underlying causes are not yet clear, but strong inter-individual differences in microbial response to dietary fiber likely play a key role [26, 27]. Therefore, the identification of the microbial taxa that mediate the beneficial effects of dietary fiber may open new avenues for individualized treatment approaches [28]. In the present study, we aimed to determine (i) whether the composition of the complex gut microbiome and metabolome (MIME) differ in lean healthy, obese healthy, and obese diabetic drug-naive type 2 diabetic patients; (ii) whether the effects of inulin on glucose tolerance and insulin sensitivity can be explained, at least in part, by the response of the gut microbiota to inulin intervention; and (iii) whether this response can be predicted from the initial MIME signature.

## Material and methods

The current study was performed within the framework of the TRIEMA project: Treatment of Insulin Resistance by Modification of Gut Microbiota (ClinicalTrials.gov Identifier: NCT03710850). The first study from the project has been already published [24].

### Study design and population

#### Observational study

Forty-nine newly diagnosed patients with pre/diabetes (DM: BMI >25, fasting glycemia >5.6 mM, and/or 2hOGTT glycemia >7.8 mM), 66 metabolically healthy overweight/obese (OB: BMI >25) and 32 lean healthy (LH: BMI <25) subjects were screened and enrolled in the cross-sectional case-control study. A clinical visit was scheduled after enrollment. Volunteers were examined after a 12-h overnight fast; blood and urine samples were collected; a clinical examination, bioimpedance analysis, and oral glucose tolerance test (OGTT, 75 g glucose) were performed. A prospective 3-day dietary record and stool samples were collected from each participant. Dietary records and stool samples were obtained no longer than a week after the clinical visit.

#### Prospective study

Twenty-seven patients (DM) were then enrolled in a one-arm, non-controlled intervention study in which they were fed 10 g of inulin daily for 3 months. The sample size determination for the intervention study was calculated for the primary outcome, glucose disposal (GD). According to GD, standard deviations ranged from 1.8 to 2.5 mg/kg/min in both insulin-sensitive and insulin-resistant individuals, with high insulin levels (i.e., 80 mU/m^2^) showing less variability with SD up to 0.51 [29]. We anticipate that participants will respond individually to the intervention, and we will divide them into tertiles (responders, neutral, and non-responders). If we consider a difference between changes of 20% (i.e., ~1.5 mg/kg/min) to be significant to have 90% power to detect a difference at the 0.05 alpha level, we must have 6 subjects in each group. To account for dropouts or incomplete data, we aimed to have at least 9 subjects in each group (i.e., responders vs. non-responders). Baseline and post-intervention examinations were identical to those described above. In addition, indirect calorimetry and a two-step glucose clamp (10 and 80 mIU/m^2^ BSA insulin dose) were performed [30]. Insulin sensitivity (IS) of adipose tissue was expressed as the change in non-esterified fatty acids (NEFA) and plasma glycerol levels from baseline to the steady state of the first step of the clamp, whereas IS of skeletal muscle was expressed as space-corrected glucose infusion rate per kg fat-free mass (Mcor mg/kg FFM/min) and metabolic clearance of glucose divided by steady-state insulinemia (MCR/I, ml/kg FFM/min) at the steady state of the second step. Detailed calculations are described in Supplementary Material. All participants signed an informed consent before enrollment in each respective study. The research protocol was approved by the Ethics Committee of University Hospital Kralovske Vinohrady (EK-VP /26/0/2017) in accordance with the Declaration of Helsinki. The study was registered under NCT03710850.

### Gut microbiome analysis

DNA from stool samples was isolated using the QIAmp PowerFecal DNA Kit (Qiagen, Hilden, Germany), and the V4 region of the bacterial 16 S rRNA gene was amplified by PCR. Sequencing was performed using the Miseq reagent kit V2 with a MiSeq instrument (Illumina, Hayward, CA, USA). The raw sequences were processed using a DADA2 Amplicon Denoiser [31].

### Short-chain fatty acids (SCFA) in plasma

SCFA were analyzed in plasma by LC-MS according to a method described before [32].

### Volatile compounds (VOCs) analysis in feces

Volatile fingerprinting of fecal samples was performed using an Agilent 7890B gas chromatograph (Santa Clara, California, USA) coupled to a Pegasus 4D time-of-flight mass spectrometer (LECO, Geleen, The Netherlands). Data acquisition and initial data processing were performed using instrumental SW ChromaTOF by LECO.

### NMR analyses

Serum samples (after protein precipitation) were measured on a 600 MHz Bruker Avance III spectrometer (Bruker BioSpin, Rheinstetten, Germany) equipped with a 5 mm TCI cryogenic probe head. The concentrations of individual metabolites, identified by comparison of proton and carbon chemical shift with the HMDB database, were expressed as PQN [33] normalized intensities of corresponding signals in CPMG spectra. The list of quantified metabolites with corresponding ^1^H and ^13^C chemical shifts is given in Table S1. The representative ^1^H NMR spectrum is shown in Fig. S1.

### Statistics

The statistical analyses were performed using R software packages and in-house scripts [34]. The microbiome and VOCs data were treated as compositional (proportions of total read count in each sample or proportion of the total area of selected masses), and before all statistical analyses, the data were transformed by centered log-ratio (clr) transformation with a multiplicative simple replacement for handling zero values. According to their abundance and prevalence, the bacteria were classified as “core microbial taxa” when fulfilling the following conditions, i.e. abundance of >0.1% and prevalence of >75% at least in one experimental group. Other microbial taxa were classified as rare.

All methods are described in detail in Supplementary Material.

## Results

### Observational study: clinical characteristics

The clinical characteristics of the study participants are shown in Table 1. As expected, the groups differed in terms of glycemic indices, insulin sensitivity, and beta cell function. Biomarkers of lipid metabolism were significantly elevated in both the OB and DM groups compared with LH.

**Table 1:** Group characteristics for lean (LH), obese (OB) and persons with pre/diabetes (DM).

	LH	DM	OB	K–W test	DMCT		
					LH vs OB	LH vs DM	OB vs DM
**General characteristics**							
Sex (F/M)	16/16	26/23	47/19				
Weight (kg)	74.8 [23.1]	99.5 [17.4]	87.2 [25.8]	˂0.05	˂0.001	˂0.001	˂0.01
Age (years)	30.9 [11.0]	58.3 [13.1]	51.3 [14.2]	˂0.05	˂0.001	˂0.001	n.s.
BMI (kg/m^2^)	23.0 [4.0]	34.9 [9.1]	30.8 [6.6]	˂0.05	˂0.001	˂0.001	˂0.05
WHR	0.8 [0.1]	1.0 [0.1]	0.9 [0.1]	˂0.05	˂0.001	˂0.001	˂0.05
**Body composition**							
Fat (kg)	14.2 [4.8]	39.5[22.3]	32.9[14.7]	˂0.05	˂0.001	˂0.001	n.s.
FFM (kg)	56.5 [22.5]	61.3[14.8]	51.9[17.3]	˂0.05	n.s.	n.s.	˂0.05
TBW (kg)	41.4 [16.5]	44.9[10.8]	38.0[12.6]	˂0.05	n.s.	n.s.	˂0.05
**Macronutrient intake**							
Total energy (kcal/day)	2101[1583]	2017[879]	1777[555]	n.s.	N/A	N/A	N/A
Proteins (g/day)	81 [29]	82 [33]	72 [28.5]	n.s.	N/A	N/A	N/A
Lipids (g/day)	83 [49]	79 [40]	65 [35.5]	˂0.05	˂0.05	n.s.	n.s.
Carbohydrates (g/day)	232 [98]	207 [96]	197 [73.5]	n.s.	N/A	N/A	N/A
Dietary fiber (g/day)	18 [19]	16 [9]	15 [7.5]	n.s.	N/A	N/A	N/A
**Glucose metabolism**							
Fasting glucose (mmol/l)	4.8 [0.3]	5.9 [0.8]	5.3 [0.6]	˂0.05	˂0.001	˂0.001	˂0.001
2 h OGTT glucose (mmol/l)	5.7 [1.1]	8.9 [3.1]	6.4 [1.6]	˂0.05	n.s.	˂0.001	˂0.001
AUC for OGTT glucose (mmol/l × 120 min^−1^)	254 [114]	499 [282]	239 [150]	˂0.05	n.s.	˂0.001	˂0.001
AUC for OGTT insulin (mIU/l × 120 min^−1^)	3890[2707]	8948[6596]	6453[4122]	˂0.05	˂0.01	˂0.001	˂0.05
Insulin (mIU/l)	4.0 [2.7]	15.9 [8.6]	9.5 [5.7]	˂0.05	˂0.001	˂0.001	˂0.001
C-peptide (pmol/l)	233 [97]	769 [357]	5.3 [0.6]	˂0.05	˂0.001	˂0.001	˂0.01
HbA1c (mmol/mol)	32 [2]	38 [7]	6.4 [1.6]	˂0.05	˂0.001	˂0.001	˂0.001
Matsuda index	10.2 [6.4]	2.0 [1.7]	4.0 [3.4]	˂0.05	˂0.01	˂0.001	˂0.001
Insulinogenic index	0.8 [0.7]	0.8 [1.0]	1.1 [1.0]	˂0.05	n.s.	n.s.	n.s.
Oral disposition index	6.7 [4.9]	1.9 [1.2]	4.9 [5.7]	˂0.001	n.s.	˂0.001	˂0.001
Beta cell index	163 [134]	45 [25]	108 [145]	˂0.001	n.s.	˂0.001	˂0.001
TyG index	0.51 [0.67]	1.54[0.59]	1.01[0.60]	˂0.05	˂0.001	˂0.001	˂0.01
**Lipid metabolism**							
Total cholesterol (mmol/l)	4.30 [1.09]	5.01 [1.23]	5.15 [1.24]	˂0.05	˂0.01	˂0.05	n.s.
HDL-C (mmol/l)	1.67 [0.47]	1.26 [0.30]	1.39 [0.56]	˂0.05	˂0.05	˂0.001	n.s.
LDL-C (mmol/l)	2.37 [1.15]	3.05 [1.40]	3.06 [1.16]	˂0.05	˂0.001	˂0.05	n.s.
Triacylglycerols (mmol/l)	0.69 [0.52]	1.53 [0.93]	1.10 [0.71]	˂0.05	˂0.001	˂0.001	˂0.05
**Inflammatory markers**							
CRP (mg/l)	0.7 [0.9]	3.3 [4.5]	2.3 [4.0]	˂0.05	˂0.001	˂0.001	n.s.
**Stool characteristics**							
pH in feces	7.26 [0.67]	7.04 [0.52]	7.27 [0.50]	n.s.	N/A	N/A	N/A
dry mass (%)	25.1 [8.9]	24.5 [9.9]	23.0 [6.9]	n.s.	N/A	N/A	N/A

Data were given as median [71].

*AUC* area under the curve during oral glucose tolerance test, *BMI* body mass index, *CRP* C-reactive protein, *DMCT* Dunn’s multiple comparison test, *FFM* fat-free mass, *HDL-C* high-density lipoprotein–cholesterol, *HbA1* glycated hemoglobin, *K–W* Kruskal–Wallis test, *LDL-C* low-density lipoprotein–cholesterol, *N/A* not applicable, *ns* not significant, *TyG index* ln (fasting triglyceride × fasting glucose)/2; *TBW* total body water, *WHR* waist-hip ratio. Insulinogenic index (ΔINS 0-30/ΔGLU 0-30), *ISI-M* Matsuda–deFronzo index; oral disposition index (IGI*ISI); beta cell index (iAUC_insulin_/iAUC_glucose_)*ISI.

*p < 0.05, **p < 0.01, ***p < 0.001.

### Observational study: fecal microbiome composition

In all samples, we found 44,332 amplicon sequence variants (ASVs) and identified 13 phyla, 30 classes, 56 orders, 104 families, and 367 genera. Considering only the ASVs, all α-diversity indices were significantly lower in OB and DM compared with LH, whereas no differences were found between the DM and OB groups (Fig. S2). When ASVs were aggregated and classified at the genus level, only the Shannon index remained significantly lower in OB and DM compared with LH (Fig. S3).

**Fig. 1:**
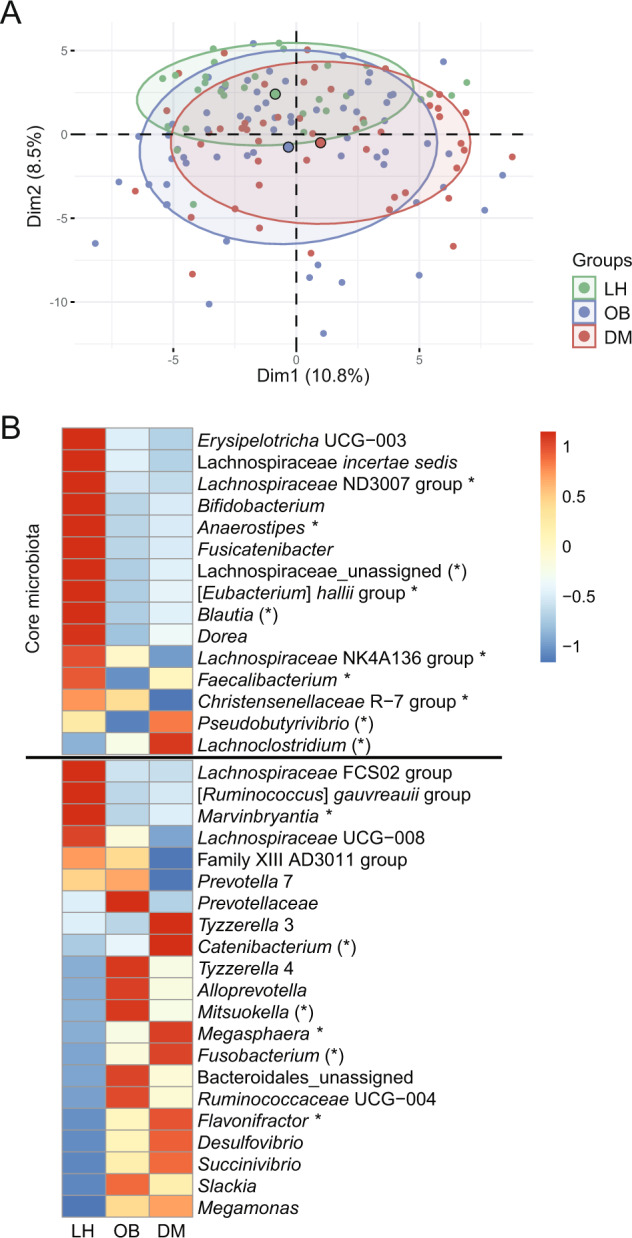
Fecal microbiome composition. **A** 2D PCA scores plot on genera level after clr transformation. The explained variance of each component is included in the axis labels. The large points represent the centroids of each group. **B** Abundances of all significant genera (FDR <0.1). Proportional data were used. Each cell then represents the mean in each group for the corresponding genera. Rows were z-scaled. Core genera are defined by the condition abundance >0.05% and prevalence >75% at least in one group. Genera marked by * are confirmed butyrate producers, and genera marked by (*) are potential butyrate producers.

At the phylum level, the microbiota composition was dominated by Firmicutes and Bacteroidetes, followed by much less abundant Actinobacteria, Proteobacteria, and Verrucomicrobia. The median abundance of all other phyla was less than 0.01%. There were no significant differences in the representation of individual phyla (Table S2). The separation of individual samples at the genus level is visualized in Fig. 1A. Multivariable statistics revealed significant differences in β-diversity (*p* ≤ 0.001), and pairwise analysis confirmed significant differences between OB vs. LH (*p* ˂ 0.001) and DM vs. LH (*p* < 0.001), but not between DM and OB. Using univariable analysis, we identified 37 taxa that had significantly different abundance among groups; 15 of them met the criteria of “core” microbiota, i.e., an abundance of >0.05% and a prevalence of >75% in at least one group (Fig. 1B and Table S3), accounting for 45% of all core genera. Thirteen core genera were more abundant in LH compared to the other two groups, while *Pseudobutyrivibrio* and *Lachnoclostridium* were enriched only in DM. Confirmed butyrate producers, i.e., *Anaerostipes, Eubacterium halii, Faecalibacterium, Christensenellaceae R-7 group*, were more abundant in the core microbiota LH than in the core microbiota OB or DM. Most of the taxa enriched in DM and/or OB belong to the “non-core” taxa. Among them, potentially harmful genera were identified (*Fusobacterium, Megasphera,* and *Desulfovibrio*). Significant positive correlations were found between *Fusobacterium* abundance and C-peptide concentration in all groups. The common or unique taxa specific to the groups are shown in Fig. S4.

The discrimination of the groups as a function of microbiome composition was investigated using a machine learning approach (LASSO regression model). This model, which has an accuracy of 51% and a sensitivity of 66% (LH), 50% (OB), and 43% (DM), does not reliably classify LH, OB, and DM (Fig. S5). When we grouped OB and DM, the accuracy of the model increased to 75% and the sensitivity to 65% (LH) (Fig. S6).

### Observational study: fecal metabolome

**Fig. 2:**
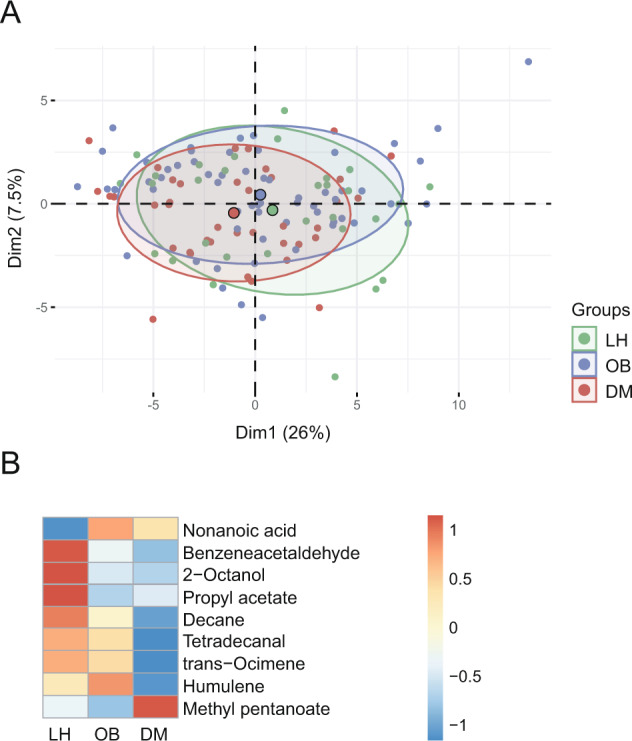
Fecal metabolome composition. **A** 2D PCA scores plot on VOCs abundances after clr transformation. Only VOCs meeting condition AUC_x,p_ ≥ 0.1% AUC_total,p_ are shown. The explained variance of each component is included in the axis labels. The large points represent the centroids of each group. **B** Abundances of significant metabolites. Proportional data were used. Each cell then represents the median in each group for the corresponding metabolite. Rows were z-scaled.

In the fecal metabolome, we identified 185 different VOCs. Within this subset, 113 VOCs were of very low abundance (˂0.1%), 54 VOCs each accounted for 0.1–1% of the total, 12 VOCs accounted for 1–5% of the total, and six were very abundant (>5%). The separation of individual samples is visualized in Fig. 2. Multivariable statistics revealed significant differences in β-diversity (*p* = 0.0017). The pairwise analysis confirmed significant differences between the DM vs. LH groups (*p* < 0.01) and OB vs. LH (*p* < 0.05), but not between DM and OB.

Univariable analysis followed by effect size analysis revealed ten VOCs with significantly different abundance between groups (FDR *p* ≤ 0.1) (Fig. 2B and Table S4). Nonanoic acid was more abundant, while all other compounds, including SCFA esters, were less abundant in the OB and DM groups compared to LH. Only methyl pentanoate showed an opposite pattern in the DM and OB groups (DM>LH = OB) (Fig. S7). Nonanoic acid correlated positively with the TyG index in all groups.

A LASSO model created for the classification of tested subjects into three categories (LH vs OB vs DM) achieved only 52% accuracy and only 48% (LH), 54% (OB), and 53% (DM) sensitivity (Fig. S8). When we combined subjects from OB and DM into one category, classification accuracy increased to 80.5%, but sensitivity remained low at 52% (Fig. S9).

### Observational study: serum/plasma metabolome

To determine the composition of the serum metabolome, we used an untargeted NMR approach and LC-MS analyzes that allows accurate determination of SCFA concentration in plasma. In total, we identified 35 quantified analytes by NMR and nine SCFAs by LC-MS, only acetate/acetic acid was identified by both methods. PERMANOVA analysis suggested the separation of the groups, and subsequent pairwise tests revealed significant differences (*p* ≤ 0.001) in serum metabolome composition between all compared pairs.

**Fig. 3:**
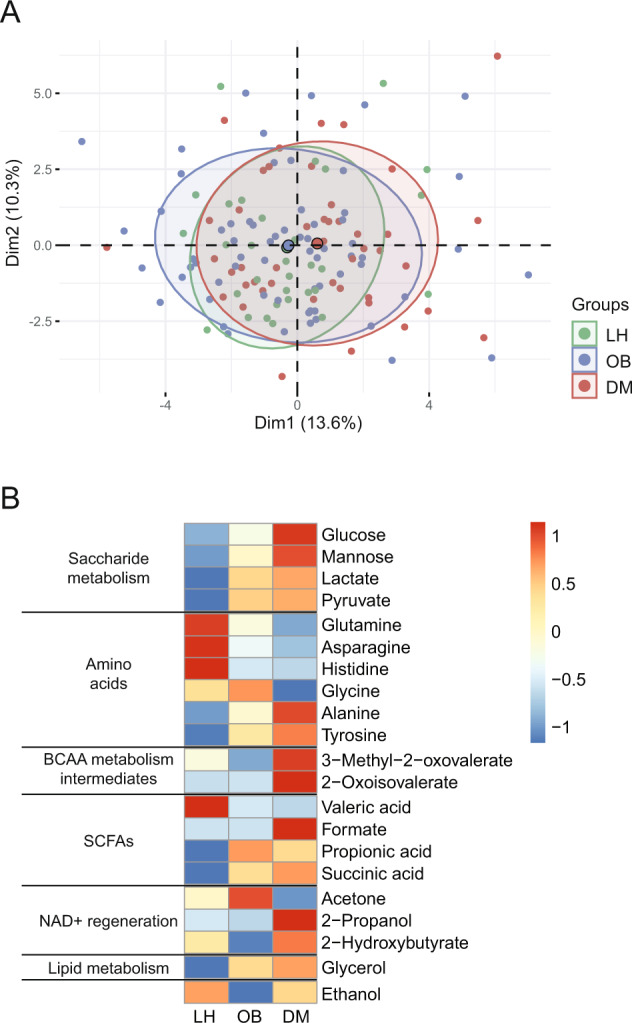
Serum metabolome composition. **A** 2D PCA scores plot. The explained variance of each component is included in the axis labels. The large points represent the centroids of each group. **B** Abundances of significant metabolites. Each cell then represents the median in each group for the corresponding metabolite. Rows were z-scaled.

The univariable analysis identified 21 metabolites that were significantly different in abundance between groups (Fig. 3B and Table S5). Based on the univariable analysis, we identified LH, OB, and DM-specific groups of serum metabolites. For most metabolites, the DM and LH groups represented the opposite poles of the abundance spectra, with OB closer to the DM group. All three groups differed in serum concentrations of intermediates of saccharide metabolism (glucose, lactate, and mannose) and two amino acids (AA) (glutamine, alanine). The concentration of seven compounds, including three SCFA (propionic acid, succinic acid, valeric acid), two AA (tyrosine, histidine), and glycerol was comparable at OB and DM, but differed from LH. Six compounds, including two branched-chain amino acid (BCAA) derivatives (2-oxoisovalerate, 3-methyl-2-oxovalerate), 2-hydroxybutyrate, acetone, 2-propanol, and formic acid, presented a specific DM-associated signature (Fig. S10).

A LASSO -model based on serum metabolome data was able to classify unknown subjects into the categories LH, OB, or DM with an accuracy of 74% and a sensitivity of 90% (LH), 72% (OB), or 65% (DM) (Fig. S11). When we grouped subjects from OB and DM groups together, model accuracy increased to 89% and sensitivity (LH) increased to 88% (Fig. S12). None of the models selected glucose as a key discriminant.

### Observational study: integrative analysis

We further investigated whether a combination of all variables would allow better classification between groups. With this integrated LASSO model, an unknown subject could be assigned to one of the three groups (LH, OB, and DM) with an accuracy of 77% and a sensitivity of 88% (LH), 79% (OB), and 66% (DM), respectively. LASSO coefficients included five variables from the microbiome dataset, one variable from the fecal metabolome dataset, and nine variables from the serum metabolome dataset (Fig. S13). When we constructed the LASSO model only for two groups (LH vs. OB + DM), we were able to classify an unknown subject with 91% accuracy and 89% sensitivity. Ten microbes, five fecal VOCs, and 11 serum metabolites contributed to the discrimination between groups (Fig. S14).

**Fig. 4:**
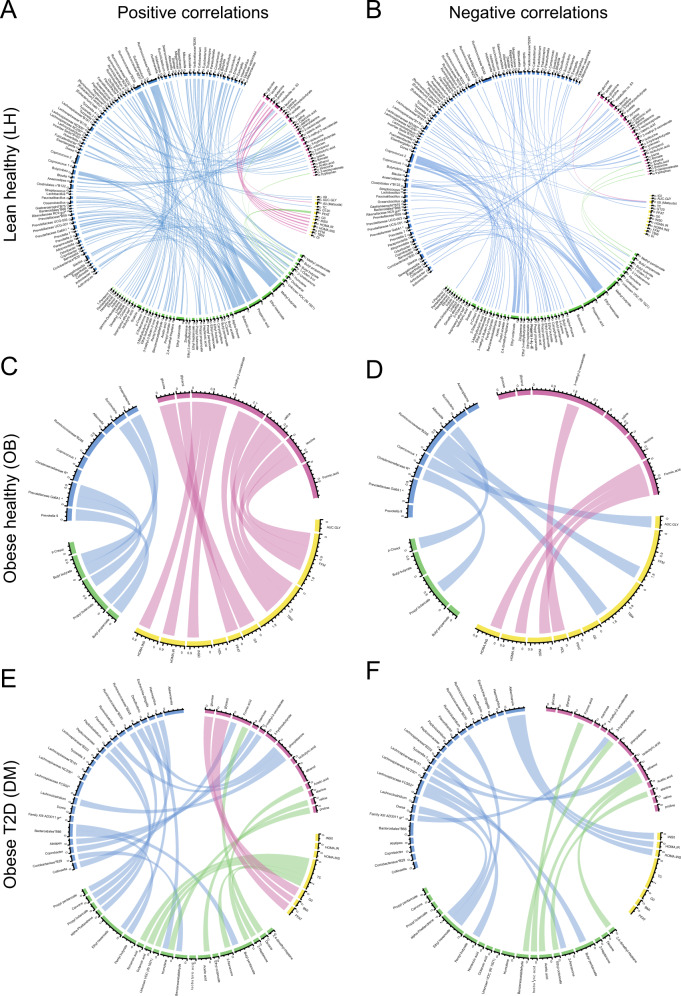
Correlation chord diagrams between variables of different datasets. Spearman correlations were calculated for each group (LH, OB, DM) separately. Only correlations among variables from different datasets (clinical variables, microbiome, serum, and fecal metabolome) and characterized by |ρ| > 0.5 are presented. Positive (**A**, **C**, **E**) and negative (**B**, **D**, **F**) correlations are shown separately. The colors on the circuit code individual datasets, the color of the edges corresponds to one of the datasets that are linked by the edge. Blue: microbiome; green: fecal metabolome; yellow: clinical variables; violet: serum metabolome.

Finally, we looked for a possible complex interaction between different MIME components in individual groups. Figure 4 depicts the positive and negative Spearman correlations among datasets filtered by |ρ| > 0.5; these correlations unravel differences in interaction networks within each group. In the LH group, we observed a rich network among variables both within and outside the datasets, whereas the complexity in OB and DM was much lower.

### Prospective study: effect of inulin on omics signature

**Fig. 5:**
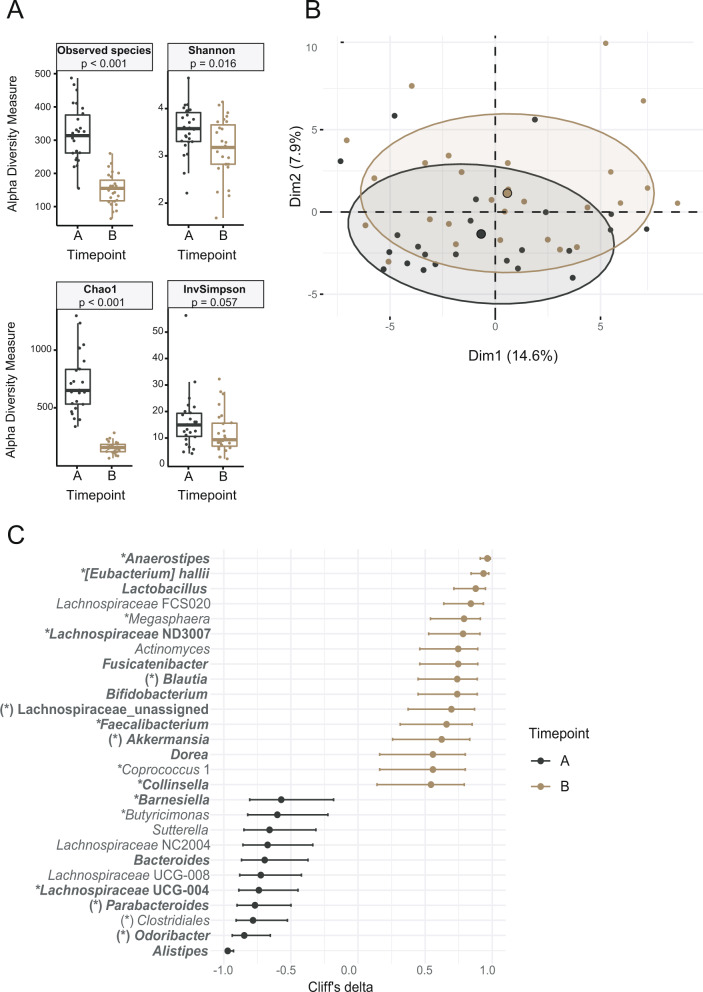
Effect of inulin on fecal microbiome composition. **A** Alpha diversity calculated on rarefied ASV data, *p* value represents the result of Wilcoxon test; **B** 2D PCA scores plot on genera level. The explained variance of each component is included in the axis labels. The large points represent the centroids of each group. **C** Biomarker bacterial genera. Prior to all calculations, data were clr transformed. Biomarkers were generated from univariable discriminant analysis (FDR ≥0.1), with effect size estimated by Cliff’s delta with a 95% confidence interval. A, time point prior to intervention; B, time point post-intervention. Core genera (bold) are defined by the condition abundance >0.05% and prevalence >75% at least in one group. Genera marked by * are confirmed butyrate producers, and genera marked by (*) are potential butyrate producers. ASV, amplicon sequence variant.

Twenty-seven newly diagnosed DM subjects participated in a three-month, single-arm, non-controlled intervention study in which they were administered inulin (10 g/day) without other antidiabetic medications and/or lifestyle interventions. No clinically significant adverse events occurred, and all subjects completed the study. The inulin intervention was associated with a significant change in microbiota composition (PERMANOVA *p* ˂ 0.001) and a significant decrease in α-diversity (Fig. 5A, B). At the phylum level, the abundance of Bacteroidetes and Proteobacteria significantly decreased, whereas the proportion of Actinobacteria and Verrucomicrobia significantly increased (Table S6). Univariable analysis revealed 28 taxa with significantly different abundance before and after inulin treatment (Fig. 5C and Table S7). The abundance of 16 bacterial taxa (genera or higher taxonomic units), including confirmed butyrate producers such as *Faecalibacterium*, *Anaerostipes*, and *Eubacterium halii* or bacteria considered beneficial such as *Lactobacillus, Bifidobacterium*, and *Akkermansia*, increased after treatment. The abundance of 12 taxa, including *Alistipes, Odoribacter*, or *Bacteroides*, decreased.

In serum and feces, inulin intake was not associated with a shift in total metabolome composition, but using univariable analysis, we identified several metabolites that were significantly different before and after the intervention. In serum, the concentration of butyric acid, propionic acid, and asparagine increased significantly, whereas the concentration of glycerol and 2-propanol decreased after inulin treatment (Fig. S15 and Table S8). In feces, three VOCs were significantly different in abundance (*p* ˂ 0.05) before and after inulin treatment, including two propionic acid esters (increased) and 1-hexanol (decreased) (Table S9). However, the significance disappeared after multiple comparisons.

### Prospective study: effect of inulin on glucose metabolism

**Fig. 6:**
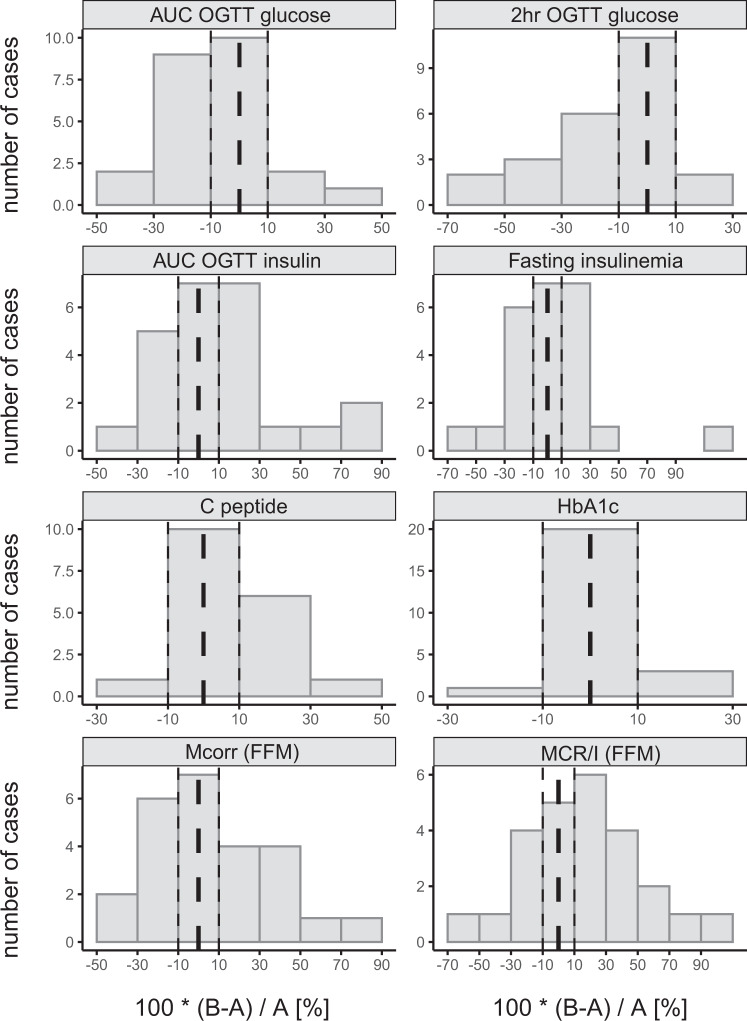
Effect of inulin supplementation on selected markers of glucose metabolism. Data are expressed as the percentual change baseline to post-intervention. A, time point prior to intervention; B, time point post-intervention. Dashed line, 0%; dotted lines, ±10% range.

Inulin intake affected markers of glucose tolerance and insulin sensitivity, but the individual response was highly variable; we observed positive, no, or negative changes for each of the variables (Fig. 6 and Table S10). In the entire intervention group, we observed a significant improvement in glucose tolerance (120 min OGTT glucose) and a trend toward a reduction in AUC for OGTT glucose and fasting glycemia. Skeletal muscle insulin sensitivity, measured by glucose clamp and expressed as MCR/I value, increased by more than 10% after the intervention compared with baseline in 14 subjects (from +11.4 to +62.4%), whereas it did not change or decrease in 13 subjects (from +4.8 to −48.7%). A similar distribution was observed for other indices of insulin sensitivity (Mcorr corrected for FFM, AUC OGTT insulin, and fasting insulinemia).

### Prospective study: predictors of the metabolic effect of inulin

Because we replicated previous findings of large inter-individual differences in metabolic responses to inulin, we sought to identify predictors of these differences. To this end, we built linear regression models for all glucose metabolism parameters studied as outcome variables, with all clinical or omics variables as predictors; we omitted variables with significant coefficients that had high leverage (Figs. S16–S19). Despite the limitations of our model, it showed several potentially interesting findings (summarized in Table 2). For example, the effect of inulin on skeletal muscle insulin sensitivity (Mcorr and MCR/I) could be predicted from pre-intervention glycemic measures. In contrast, the MIME predictors of the inulin effect were mostly not associated with pre-intervention outcome variables. Change in AUC OGTT glucose was negatively associated with an initial abundance of *Ruminiclostridium*, whereas increases in Mcorr and MCR/I were associated with higher initial serum asparagine (both parameters) and lower *Dialister* (Mcorr) or *Blautia* and *Eubacterium halii* (MCR/I). Initial serum concentrations of BCAA derivatives were positively associated with increases in AUC and 2-hour OGTT glucose. All results are summarized in Table 2.

**Table 2:** Predictors of the inulin treatment effect on glucose homeostasis parameters.

outcome	predictor	$\widehat \beta _x$	*p* val βx	$\widehat \beta _y$	*p* val βy	*R*^2^
AUC OGTT glucose	*Ruminiclostridium*	−41.60	0.015	−0.11	0.236	0.393
	*Lachnospiraceae_incertae sedis*	40.37	0.015	−0.18	0.044	0.249
	*Lachnoclostridium*	35.83	0.033	−0.16	0.083	0.317
	3-methyl-2-oxovalerate	37.78	0.018	−0.18	0.044	0.326
	alanine	36.28	0.024	−0.19	0.040	0.287
	ethanol	−51.31	0.001	−0.21	0.008	0.501
2 h OGTT glucose	AUC OGTT insulin	1.00	0.027	−0.03	0.876	0.286
	fasting insulinemia	0.91	0.037	−0.10	0.536	0.181
	HOMA INS	0.88	0.046	−0.12	0.487	0.215
	*Eubacterium halii group*	0.99	0.032	−0.17	0.321	0.280
	3-methyl-2-oxovalerate	1.08	0.012	−0.09	0.585	0.287
	3-hydroxyisobutyrate	0.98	0.024	−0.14	0.392	0.278
	2-oxoisocaproate	0.93	0.033	−0.12	0.462	0.229
	pyruvate	0.93	0.034	−0.09	0.580	0.245
	indole	1.33	0.002	−0.11	0.465	0.350
	tridecanol	1.17	0.008	−0.13	0.426	0.300
	ç-Dodecalactone	1.13	0.012	−0.18	0.276	0.269
	methyl heptenone	1.05	0.020	−0.16	0.360	0.217
	2-undecanone	1.05	0.021	−0.16	0.341	0.240
	methyl butanal	1.02	0.024	−0.11	0.527	0.214
MCR/I (FFM)	ISI (Matsuda)	0.01	0.005	−0.67	0.008	0.313
	AUC OGTT insulin	−0.01	0.005	−0.53	0.017	0.291
	2 hr OGTT insulinemia	−0.01	0.024	−0.45	0.051	0.251
	HOMA INS	−0.01	0.027	−0.57	0.029	0.208
	fasting insulinemia	−0.01	0.030	−0.56	0.032	0.269
	HOMA IR	−0.01	0.036	−0.55	0.035	0.205
	IGI	−0.01	0.045	−0.40	0.080	0.189
	*Blautia*	−0.01	0.027	−0.22	0.329	0.222
	*[Eubacterium] hallii group*	−0.01	0.030	−0.16	0.451	0.180
	asparagine	0.01	0.011	−0.31	0.121	0.230
∆ Mcorr (FFM)	ISI (Matsuda)	1.02	0.001	−0.53	0.025	0.474
	AUC OGTT insulin	−0.90	0.002	−0.31	0.132	0.335
	HOMA INS	−0.90	0.003	−0.38	0.080	0.318
	Fasting insulinemia	−0.88	0.003	−0.37	0.090	0.326
	HOMA IR	−0.86	0.006	−0.38	0.091	0.243
	IGI	−0.75	0.009	−0.20	0.367	0.243
	2 h OGTT insulinemia	−0.66	0.029	−0.24	0.293	0.201
	*Dialister*	−0.58	0.038	−0.01	0.979	0.172
	*Phascolarctobacterium*	0.55	0.048	0.08	0.705	0.203
	asparagine	0.75	0.011	−0.26	0.239	0.210

The data shown in the table are derived from the linear regression model described by the equation Y^(B)^ – Y^(A)^ = β_0_ + β_Y_Y^(A)^ + β_X_X^(A)^ + ε, where Y^(A)^ stands for outcome variable at time A; Y^(B)^ stands for outcome variable at time B, B > A; X^(A)^ stands for a standardized variable at time A representing in each model any single clinical, metabolome or microbiome variable; β_x_, β_y_ are model coefficients; ε stands for random error. Fecal metabolites were filtered by the condition Σ AUC_x_ ≥0.1% Σ AUC_total_ across all samples; bacteria were filtered by the condition median abundance ≥0.1% of the total Σ of bacteria across all samples.

*HbA1C* glycosylated hemoglobin, *Mcorr* glucose disposal space corrected and adjusted to fat-free mass, *MCR/I* metabolic clearance rate of glucose space corrected and adjusted to fat-free mass divided by steady-state insulinemia, *OGTT* oral glucose tolerance test, *R*^2^ proportion of variation in y explained by the predictors obtained using bootstrapping (50 iterations).

## Discussion

Our main findings are: (i) obesity is the dominant factor determining the MIME signature, whereas glycemic status has a lesser additional influence; (ii) the metabolic response to inulin supplementation in individuals with newly diagnosed prediabetes/diabetes is highly variable but can be predicted, at least in part, from baseline clinical characteristics and MIME signatures. Indeed, more insulin-resistant individuals with poorer glycemic indices and elevated circulating BCAA derivatives and fecal indole and p-cresol are less likely to respond to inulin supplementation.

### Observational study: gut microbiome and metabolome

Obesity is a prominent risk factor for the development of type 2 diabetes. Numerous studies have identified groups of bacterial taxa that are enriched or depleted in obesity and type 2 diabetes, and despite considerable heterogeneity in the results, some common observations have been noted. First, type 2 diabetes is associated with the depletion of potentially beneficial bacteria rather than the presence of some dominant potentially harmful bacteria. Second, the abundance of butyrate producers and the functional potential for butyrate production is reduced in type 2 diabetes [10, 20, 35]. Third, the diversity of the microbiota is lower in diseased individuals compared with healthy controls [6, 36].

Some of our results are consistent with the above, whereas others are contradictory. In contrast to the results of Wu [10], the change in the composition of the gut microbiota in our study was not related to glycemic status but mainly to obesity. The dominant butyrate producers, such as *Faecalibacterium*, *Anaerostipes*, *Eubacterium halii*, or *Blautia* were significantly less abundant in the microbiota of DM and OB, but we did not detect lower SCFA concentrations in either feces or serum. In contrast, MCFA, nonanoic and decanoic acids were elevated in OB and DM. MCFA can originate from dietary sources [35], but also from microbial or yeast fermentation [37]. SCFA and MCFA have different immunomodulatory properties; whereas SCFA attenuate inflammation, MCFA have the opposite effect [35, 38, 39]. In addition, MCFA may enhance intestinal permeation because of their physicochemical properties as anionic surfactants [40]. Based on these findings, we might suggest that it is not the lower level of SCFA but the increased level of MCFA in the lumen that contributes to the complications associated with obesity, such as impaired intestinal barrier function or chronic low-grade inflammation.

### Observational study: serum metabolome

The serum metabolome signature of obesity and diabetes overlapped greatly in the study. Compared to lean subjects, both the OB and DM signatures follow the same direction and differ only in magnitude. The “adiposity signature,” which is similar in both OB and DM, includes SCFA (succinic and propionic acid increased, while valeric acid decreased), aromatic AA tyrosine (increased), and two other AA (histidine and asparagine, decreased). The concentration shift of five other metabolites, i.e., intermediates of saccharide metabolism (glucose, lactate, and mannose, increased) and AA glutamine and alanine, follows the concordant direction to LH, but there is a significant difference among all three groups. Six metabolites are specific for DM. This signature consists of three BCAA derivatives, formic acid, 2-hydroxybutyrate, acetone, and 2-propanol.

Our findings are consistent with previously published observations [41, 42]. Some signature metabolites could be attributed to altered saccharide metabolism in obesity and diabetes, such as glucose, mannose, and lactate. 2-propanol, acetone, and 2-hydroxybutyrate might be related to NADH/NAD+ redox imbalance, which has been proposed as one of the features of T2D [43].

Some other signature metabolites, i.e., SCFA and BCAA, are located at the interface between the host and microbiota. SCFA in serum have not previously been described as components of an obesity-related serum signature, probably because of the analytical difficulties associated with their determination in serum. They are exclusively microbial products, some of which (circulating butyric acid and propionic acid) have been associated with beneficial effects [44]. Elevated circulating BCAAs have been associated with insulin-resistance conditions such as obesity, diabetes [45], and even cancer [46]. For mammals, BCAAs are essential and must be supplied from external sources. Recent research has deciphered the importance of the gut microbiota in modulating the availability of many necessary compounds, including BCAA, to the host [47].

### Inulin intervention and the effects on microbiota composition and performance

Three months of regular consumption of 10 g inulin/day was associated with a significant shift in the composition of the microbiota, characterized by a marked increase in potentially beneficial bacteria, many of which are capable of butyrate production [48]. At the same time, several bacterial taxa were depleted, such as those associated with the fermentation of proteins [49, 50]. This observation is largely consistent with previously published reports [51, 52].

We did not detect a significant shift in the composition of the fecal metabolome, although there was a non-significant trend toward an increase in SCFA esters content. Participants were asked not to change their dietary habits, and the only difference before and after the intervention was the amount of inulin consumed. This change could primarily increase the production of SCFA, but these compounds are readily utilized by other microbes or colonocytes at the site of their production, and only about 5% of SCFA are excreted in the feces [53]. A small fraction of SCFA from the intestine may enter the bloodstream, and indeed we observed a significant increase in serum butyric and propionic acid concentrations at the end of the intervention. Muller et al. [54] have previously reported that it is not fecal but circulating SCFA, particularly butyrate, that can provide a link between the gut microbiota and whole-body insulin sensitivity. SCFA are ligands of the G protein-coupled receptors GPR41 and GPR43, which are expressed in many tissues, including adipose tissue and skeletal muscle [55, 56]. Animal studies have shown that oral administration of SCFA or intravenous infusion improves insulin sensitivity [54].

### Predicting the individual effect of an inulin intervention

The increasing understanding of the role of the microbiome in host physiology opened new avenues for research focused on the possibility of predicting the outcome of a given intervention based on the individual MIME setting. Clinically relevant results have been obtained in cancer research, e.g., the success of therapy with Anti-programmed Cell Death Protein-1 (PD−1) has been shown to depend significantly on the baseline composition of the patient’s gut microbiota [57–60]. MIME has also been successfully used to predict the response of IBD patients to a low FODMAP diet [61] or anti-TNF treatment [62], the efficacy of synbiotic treatment of gastrointestinal disease in children [63], or the prediction of the clinical outcome of bariatric surgery [64]. The gut microbiota may serve as a biomarker for selecting the most effective drugs for the treatment of rheumatoid arthritis [65], and gut bacterial signatures have even been described to characterize the diagnosis and predict treatment outcomes in bipolar depression [66].

Inulin-type dietary fiber is thought to alleviate several features of metabolic syndrome; however, results from human studies are inconsistent. A recent systematic review [67], which included 33 RCTs, showed that inulin intake (average 11 g/day) significantly reduced blood glucose, total cholesterol, and TAG in individuals with prediabetes and diabetes. However, a common feature of all included studies was the wide heterogeneity of individual responses to treatment, making clear dietary recommendations difficult. Therefore, we sought to identify factors that would allow a personalized assessment of the efficacy of inulin treatment. We found that patients with a profile suggestive of less impaired glucose homeostasis were likely to improve metabolically. In addition, we identified several other potential predictors that were not dependent on pre-intervention glycemic indices, including lower serum BCAA derivatives (3-methy-2-oxovalerate, 2-oxoisocaproate), serum 3-hydroxyisobutyrate (product of NADH oxidation), fecal indole, and/or various bacteria (*Ruminiclostridium, Lachnoclostridium, Eubacterium halii*, etc.), which could allow a more accurate prediction of inulin intervention outcomes. In the prediabetes phase, patients are often advised to change their lifestyle and diet. Despite initial adherence to advice, outcomes may be highly variable, and patients who have failed despite their best efforts may be demotivated to adhere to further recommendations. The tool of predicting the individual appropriateness of a particular intervention, in this case, the administration of inulin, would help personalize treatment so that it has a higher chance of success in potential responders and does not expose potential non-responders to repeated failures.

### Strengths and limitations of the study

There are several strengths of the study. First, the DM group included only participants with newly diagnosed type 2 diabetes prior and/or concomitant treatment, thus excluding confounding effects of antidiabetic drugs on the effects of inulin. Second, we did not rely solely on the measurement of fecal SCFA as the only indicator of SCFA production in the colon, but used a highly sensitive LC-MS method that allows its quantification in serum. Third, we evaluated the complex effects of the inulin intervention using a multi-omics approach. Nevertheless, the study is limited by several factors. First, we were able to include only a limited number of subjects, and the results were not validated in an independent cohort. For this, the results were internally validated by permutation tests. Second, the lean healthy subjects differed from the OB or DM groups by age, because obesity and associated comorbidities are more common in older populations. Age is one of the external factors affecting microbiota composition, but this is especially true for very young children or the elderly (over 70 years of age). In adolescence and adulthood, the composition of the microbiome is remarkably stable in terms of diversity indices, PCA metrics, or representation of selected taxa [68–70]. Therefore, we believe that the age difference in our population did not result in a significant bias. Third, we did not control dietary intake during the prospective intervention study with inulin because we did not want to further burden participants and increase the risk of dropping out of the study, but all participants were explicitly asked to maintain their usual dietary habits. An indirect measure of adherence to the habitual diet may be the BMI of participants, which did not change significantly during the intervention period. Finally, the prospective study design was a single-arm, non-controlled intervention study, so the causality of the effect of inulin on metabolic outcomes cannot be inferred. The small number of participants in the prospective study did not allow us to build more complex models to account for possible synergies among predictors. Because the study aimed to explore predictors, and we found several novel biomarkers that predict response to inulin treatment, these will need to be validated on a larger scale in future studies.

In summary, we showed that the gut microbiota and metabolome profiles in OB and DM differed from those of lean healthy individuals, whereas the differences between OB and DM were less pronounced. We identified several omics-derived biomarkers that may play a central role in the development of obesity-associated metabolic changes. In patients with newly diagnosed pre/diabetes, we observed substantial inter-individual variability in the effects of inulin on glucose homeostasis and identified several predictors of treatment response. If replicated in further studies with other populations, the identified predictors could facilitate the estimation of inulin intervention outcomes, paving the way for the concept of personalized dietary management of early diabetes.

## Supplementary information

Supplemental Material

## Data Availability

The sequencing data were available at the SRA database under the accession number PRJNA823864. The other datasets generated and analyzed during the current study are available from the corresponding author on request.

## References

[1] Cheng HL, Medlow S, Steinbeck K (2016). The health consequences of obesity in young adulthood. Curr Obes Rep..

[2] Turnbaugh PJ, Ley RE, Mahowald MA, Magrini V, Mardis ER, Gordon JI (2006). An obesity-associated gut microbiome with increased capacity for energy harvest. Nature..

[3] Cornejo-Pareja I, Munoz-Garach A, Clemente-Postigo M, Tinahones FJ (2019). Importance of gut microbiota in obesity. Eur J Clin Nutr.

[4] Lim YY, Lee YS, Ooi DSQ (2020). Engineering the gut microbiome for treatment of obesity: a review of current understanding and progress. Biotechnol J..

[5] Qin J, Li Y, Cai Z, Li S, Zhu J, Zhang F (2012). A metagenome-wide association study of gut microbiota in type 2 diabetes. Nature..

[6] Nuli R, Cai J, Kadeer A, Zhang Y, Mohemaiti P (2019). Integrative analysis toward different glucose tolerance-related gut microbiota and diet. Front Endocrinol.

[7] Zhou W, Sailani MR, Contrepois K, Zhou Y, Ahadi S, Leopold SR (2019). Longitudinal multi-omics of host-microbe dynamics in prediabetes. Nature..

[8] Forslund K, Hildebrand F, Nielsen T, Falony G, Le Chatelier E, Sunagawa S (2015). Disentangling type 2 diabetes and metformin treatment signatures in the human gut microbiota. Nature..

[9] Allin KH, Tremaroli V, Caesar R, Jensen BAH, Damgaard MTF, Bahl MI (2018). Aberrant intestinal microbiota in individuals with prediabetes. Diabetologia..

[10] Wu H, Tremaroli V, Schmidt C, Lundqvist A, Olsson LM, Kramer M (2020). The gut microbiota in prediabetes and diabetes: a population-based cross-sectional study. Cell Metab.

[11] Tilg H, Moschen AR (2014). Microbiota and diabetes: an evolving relationship. Gut..

[12] Zhang X, Shen D, Fang Z, Jie Z, Qiu X, Zhang C (2013). Human gut microbiota changes reveal the progression of glucose intolerance. PLoS ONE.

[13] Bhute SS, Suryavanshi MV, Joshi SM, Yajnik CS, Shouche YS, Ghaskadbi SS (2017). Gut microbial diversity assessment of Indian type-2-diabetics reveals alterations in Eubacteria, Archaea, and Eukaryotes. Front Microbiol.

[14] Zhao L, Lou H, Peng Y, Chen S, Zhang Y, Li X (2019). Comprehensive relationships between gut microbiome and faecal metabolome in individuals with type 2 diabetes and its complications. Endocrine..

[15] Zhong H, Ren H, Lu Y, Fang C, Hou G, Yang Z (2019). Distinct gut metagenomics and metaproteomics signatures in prediabetics and treatment-naive type 2 diabetics. EBioMedicine..

[16] Wang L, Yu X, Xu X, Ming J, Wang Z, Gao B (2021). The fecal microbiota is already altered in normoglycemic individuals who go on to have type 2 diabetes. Front Cell Infect Microbiol.

[17] Letchumanan G, Abdullah N, Marlini M, Baharom N, Lawley B, Omar MR (2022). Gut microbiota composition in prediabetes and newly diagnosed type 2 diabetes: a systematic review of observational studies. Front Cell Infect Microbiol.

[18] Gaike AH, Paul D, Bhute S, Dhotre DP, Pande P, Upadhyaya S (2020). The gut microbial diversity of newly diagnosed diabetics but not of prediabetics is significantly different from that of healthy nondiabetics. mSystems.

[19] Li L, Li C, Lv M, Hu Q, Guo L, Xiong D (2020). Correlation between alterations of gut microbiota and miR-122-5p expression in patients with type 2 diabetes mellitus. Ann Transl Med.

[20] Karlsson FH, Tremaroli V, Nookaew I, Bergstrom G, Behre CJ, Fagerberg B (2013). Gut metagenome in European women with normal, impaired and diabetic glucose control. Nature..

[21] Diener C, Reyes-Escogido ML, Jimenez-Ceja LM, Matus M, Gomez-Navarro CM, Chu ND (2020). Progressive shifts in the gut microbiome reflect prediabetes and diabetes development in a treatment-naive Mexican cohort. Front Endocrinol.

[22] Ghaemi F, Fateh A, Sepahy AA, Zangeneh M, Ghanei M, Siadat SD (2020). Intestinal microbiota composition in Iranian diabetic, pre-diabetic and healthy individuals. J Diabetes Metab Disord.

[23] Chen PC, Chien YW, Yang SC (2019). The alteration of gut microbiota in newly diagnosed type 2 diabetic patients. Nutrition..

[24] Prochazkova M, Budinska E, Kuzma M, Pelantova H, Hradecky J, Heczkova M (2021). Vegan diet is associated with favorable effects on the metabolic performance of intestinal microbiota: a cross-sectional multi-omics study. Front Nutr.

[25] Colantonio AG, Werner SL, Brown M (2020). The effects of prebiotics and substances with prebiotic properties on metabolic and inflammatory biomarkers in individuals with type 2 diabetes mellitus: a systematic review. J Acad Nutr Diet.

[26] Davis LM, Martinez I, Walter J, Goin C, Hutkins RW (2011). Barcoded pyrosequencing reveals that consumption of galactooligosaccharides results in a highly specific bifidogenic response in humans. PLoS ONE.

[27] Zhao L, Zhang F, Ding X, Wu G, Lam YY, Wang X (2018). Gut bacteria selectively promoted by dietary fibers alleviate type 2 diabetes. Science..

[28] Wareham NJ. Personalised prevention of type 2 diabetes. Diabetologia. 2022;65:1796–1803. 10.1007/s00125-022-05774-7 PMC9522721 35916901

[29] Le DS, Brookshire T, Krakoff J, Bunt JC (2009). Repeatability and reproducibility of the hyperinsulinemic-euglycemic clamp and the tracer dilution technique in a controlled inpatient setting. Metabolism..

[30] DeFronzo RA, Tobin JD, Andres R (1979). Glucose clamp technique: a method for quantifying insulin secretion and resistance. Am J Physiol.

[31] Callahan BJ, McMurdie PJ, Rosen MJ, Han AW, Johnson AJ, Holmes SP (2016). DADA2: high-resolution sample inference from Illumina amplicon data. Nat Methods.

[32] Han J, Lin K, Sequeira C, Borchers CH (2015). An isotope-labeled chemical derivatization method for the quantitation of short-chain fatty acids in human feces by liquid chromatography-tandem mass spectrometry. Anal Chim Acta.

[33] Dieterle F, Ross A, Schlotterbeck G, Senn H (2006). Probabilistic quotient normalization as robust method to account for dilution of complex biological mixtures. Application in 1H NMR metabonomics. Anal Chem.

[34] R Core Team. R: a language and environment for statistical computing. Vienna, Austria: R Foundation for Statistical Computing. 2017.

[35] Sam QH, Ling H, Yew WS, Tan Z, Ravikumar S, Chang MW, et al. The divergent immunomodulatory effects of short chain fatty acids and medium chain fatty acids. Int J Mol Sci. 2021;22:6453. 10.3390/ijms22126453 PMC8234078 34208638

[36] Le Chatelier E, Nielsen T, Qin J, Prifti E, Hildebrand F, Falony G (2013). Richness of human gut microbiome correlates with metabolic markers. Nature..

[37] Nissen L, Samaei SP, Babini E, Gianotti A (2020). Gluten free sourdough bread enriched with cricket flour for protein fortification: Antioxidant improvement and Volatilome characterization. Food Chem.

[38] Luscombe VB, Lucy D, Bataille CJR, Russell AJ, Greaves DR (2020). 20 years an orphan: is GPR84 a plausible medium-chain fatty acid-sensing receptor?. DNA Cell Biol.

[39] Saresella M, Marventano I, Barone M, La Rosa F, Piancone F, Mendozzi L (2020). Alterations in circulating fatty acid are associated with gut microbiota dysbiosis and inflammation in multiple sclerosis. Front Immunol.

[40] Brayden DJ, Maher S, Bahar B, Walsh E (2015). Sodium caprate-induced increases in intestinal permeability and epithelial damage are prevented by misoprostol. Eur J Pharm Biopharm.

[41] Halama A, Suleiman NN, Kulinski M, Bettahi I, Hassoun S, Alkasem M (2020). The metabolic footprint of compromised insulin sensitivity under fasting and hyperinsulinemic-euglycemic clamp conditions in an Arab population. Sci Rep..

[42] Gall WE, Beebe K, Lawton KA, Adam KP, Mitchell MW, Nakhle PJ (2010). alpha-hydroxybutyrate is an early biomarker of insulin resistance and glucose intolerance in a nondiabetic population. PLoS ONE.

[43] Song J, Yang X, Yan LJ (2019). Role of pseudohypoxia in the pathogenesis of type 2 diabetes. Hypoxia.

[44] Kumar J, Rani K, Datt C (2020). Molecular link between dietary fibre, gut microbiota and health. Mol Biol Rep..

[45] Newgard CB (2012). Interplay between lipids and branched-chain amino acids in development of insulin resistance. Cell Metab.

[46] Rossmeislova L, Gojda J, Smolkova K (2021). Pancreatic cancer: branched-chain amino acids as putative key metabolic regulators?. Cancer Metastasis Rev.

[47] Gojda J, Cahova M. Gut microbiota as the link between elevated BCAA serum levels and insulin resistance. Biomolecules. 2021;11:1414. 10.3390/biom11101414 PMC8533624 34680047

[48] Le Bastard Q, Chapelet G, Javaudin F, Lepelletier D, Batard E, Montassier E (2020). The effects of inulin on gut microbial composition: a systematic review of evidence from human studies. Eur J Clin Microbiol Infect Dis.

[49] Diether NE, Willing BP. Microbial fermentation of dietary protein: an important factor in diet(-)microbe(-)host interaction. Microorganisms. 2019;7:19. 10.3390/microorganisms7010019 PMC6352118 30642098

[50] Yao CK, Muir JG, Gibson PR (2016). Review article: insights into colonic protein fermentation, its modulation and potential health implications. Aliment Pharm Ther.

[51] Vandeputte D, Tito RY, Vanleeuwen R, Falony G, Raes J (2017). Practical considerations for large-scale gut microbiome studies. FEMS Microbiol Rev.

[52] Healey G, Murphy R, Butts C, Brough L, Whelan K, Coad J (2018). Habitual dietary fibre intake influences gut microbiota response to an inulin-type fructan prebiotic: a randomised, double-blind, placebo-controlled, cross-over, human intervention study. Br J Nutr.

[53] Rechkemmer G, Ronnau K, von Engelhardt W (1988). Fermentation of polysaccharides and absorption of short chain fatty acids in the mammalian hindgut. Comp Biochem Physiol A Comp Physiol.

[54] Muller M, Hernandez MAG, Goossens GH, Reijnders D, Holst JJ, Jocken JWE (2019). Circulating but not faecal short-chain fatty acids are related to insulin sensitivity, lipolysis and GLP-1 concentrations in humans. Sci Rep..

[55] Tang C, Ahmed K, Gille A, Lu S, Grone HJ, Tunaru S (2015). Loss of FFA2 and FFA3 increases insulin secretion and improves glucose tolerance in type 2 diabetes. Nat Med.

[56] Koh A, De Vadder F, Kovatcheva-Datchary P, Backhed F (2016). From dietary fiber to host physiology: short-chain fatty acids as key bacterial metabolites. Cell..

[57] Mao J, Wang D, Long J, Yang X, Lin J, Song Y, et al. Gut microbiome is associated with the clinical response to anti-PD-1 based immunotherapy in hepatobiliary cancers. J Immunother Cancer. 2021;9:e003334. 10.1136/jitc-2021-003334 PMC8650503 34873013

[58] Fahrmann JF, Saini NY, Chia-Chi C, Irajizad E, Strati P, Nair R (2022). A polyamine-centric, blood-based metabolite panel predictive of poor response to CAR-T cell therapy in large B cell lymphoma. Cell Rep. Med.

[59] Sannicolo S, Giaj Levra M, Le Gouellec A, Aspord C, Boccard J, Chaperot L (2021). Identification of a predictive metabolic signature of response to immune checkpoint inhibitors in non-small cell lung cancer: METABO-ICI clinical study protocol. Respir Med Res.

[60] McCulloch JA, Davar D, Rodrigues RR, Badger JH, Fang JR, Cole AM (2022). Intestinal microbiota signatures of clinical response and immune-related adverse events in melanoma patients treated with anti-PD-1. Nat Med.

[61] Vervier K, Moss S, Kumar N, Adoum A, Barne M, Browne H (2022). Two microbiota subtypes identified in irritable bowel syndrome with distinct responses to the low FODMAP diet. Gut..

[62] Busquets D, Oliver L, Amoedo J, Ramio-Pujol S, Malagon M, Serrano M (2021). RAID prediction: pilot study of fecal microbial signature with capacity to predict response to anti-TNF treatment. Inflamm Bowel Dis.

[63] Tierney BT, Versalovic J, Fasano A, Petrosino JF, Chumpitazi BP, Mayer EA, et al. Functional response to a microbial synbiotic in the gastrointestinal system of children: a randomized clinical trial. Pediatr Res. 2022. 10.1038/s41390-022-02289-0. 10.1038/s41390-022-02289-0 PMC10313516 36319696

[64] Vaz M, Pereira SS, Monteiro MP (2022). Metabolomic signatures after bariatric surgery - a systematic review. Rev Endocr Metab Disord.

[65] Wei M, Chu CQ (2022). Prediction of treatment response: personalized medicine in the management of rheumatoid arthritis. Best Pr Res Clin Rheumatol.

[66] Lai J, Li A, Jiang J, Yuan X, Zhang P, Xi C (2022). Metagenomic analysis reveals gut bacterial signatures for diagnosis and treatment outcome prediction in bipolar depression. Psychiatry Res.

[67] Li L, Li P, Xu L (2021). Assessing the effects of inulin-type fructan intake on body weight, blood glucose, and lipid profile: a systematic review and meta-analysis of randomized controlled trials. Food Sci Nutr.

[68] Odamaki T, Kato K, Sugahara H, Hashikura N, Takahashi S, Xiao JZ (2016). Age-related changes in gut microbiota composition from newborn to centenarian: a cross-sectional study. BMC Microbiol.

[69] Rodriguez JM, Murphy K, Stanton C, Ross RP, Kober OI, Juge N (2015). The composition of the gut microbiota throughout life, with an emphasis on early life. Micro Ecol Health Dis.

[70] Salazar N, Gonzalez S, Nogacka AM, Rios-Covian D, Arboleya S, Gueimonde M (2020). Microbiome: effects of ageing and diet. Curr Issues Mol Biol.

[71] Feins EN, Ireland C, Gauvreau K, Chavez M, Callahan R, Jenkins KJ (2022). Pulmonary vein stenosis: anatomic considerations, surgical management, and outcomes. J Thorac Cardiovasc Surg.

