# Supplementary material for: Multi-omics signatures in new-onset diabetes predict metabolic response to dietary inulin: findings from an observational study followed by an interventional trial
Source: Nutr Diabetes. 2023 Apr 21;13:7. doi: 10.1038/s41387-023-00235-5 (PMC10121613; doi:10.1038/s41387-023-00235-5)
Supplement: Supplementary file 1 — Supplemental Material [file 41387_2023_235_MOESM1_ESM.pdf]

Supplementary Material

1 Supplementary Data

Characterization of study cohorts

Recruitment

A/ Observational study. Forty-seven newly diagnosed patients with pre/diabetes (DM: BMI>25, fasting glycemia >5.6mM and/or 2hOGTT glycemia >7.8mM), 69 metabolically healthy obese (OB: BMI>30) and 33 lean healthy (LH: BMI<25) subjects were screened and enrolled between October 2018 and October 2019 in the cross-sectional case-control study. Patients with known history of diabetes, decompensated endocrine diseases, active or past cancer, IBD and/or antibiotics use 3 months prior the examination were not enrolled. Clinical visit was scheduled after enrollment. After 12-hours overnight fast, blood samples were taken and clinical examination performed: indirect calorimetry (QuarkRMR, Cosmed, Italy), BIA (Nutriguard, Germany), oral glucose tolerance test (OGTT, 75g glucose) with blood sampling for glucose and insulin at 0, 30, 60, 90 and 120 min.

B/ Prospective study. Twenty-seven DM patients were consequently enrolled to one-arm non-controlled interventional trial with 10 g inulin for 3 months (FAN a.s., CR). The examination at baseline and after the intervention was identical with the cross-sectional study, additionally two-step (10 and 80 mIU/m^2 BSA insulin dose) was performed. Insulin sensitivity was expressed as space corrected glucose infusion rate (M_{cor} mg/kg FFM/min) and metabolic clearance of glucose divided by steady-state insulinemia (MCR/I, ml/kg FFM/min). All participants signed an informed consent prior to enrollment to each respective study. The research protocol was approved by the Ethics Committee of the 3rd Faculty of Medicine of Charles University and Ethics Committee of University Hospital Kralovske Vinohrady in accordance with the Declaration of Helsinki.

Anthropometry and clinical examination

Each subject underwent a basic medical check-up with an antropometric examination (height, weight, BMI, waist circumference and waist-to-hip ratio). Body composition was determined by bioimpedance analysis (Nutriguard-M, Data Input GmbH, Frankfurt, Germany). Resting metabolic rate was measured by indirect calorimetry and calculated by Harris-Benedict equation.

Dietary assessment

Three-day prospective record supervised by trained dietitian was used to assess macronutrient and fiber composition of the diet. Each participant filled in a prospective record, where dietary data from three typical days were collected (two working days, one weekend day). Volunteers were educated and instructions were given for portion sizes estimation and recording foods in sufficient detail to obtain an accurate estimate of consumed portions and were given portion estimation guide as a reference. Moreover, examples of complete and incomplete diaries were explained to show how to appropriately record the intake. After the collection, the records were retrospectively checked by independent researcher. USDA database was used for assessment of food composition, NutriServis PROFI, CR, program was used for dietary intake calculations. Daily intake of carbohydrates, lipids, proteins and dietary fiber were calculated separately.

Laboratory analysis

Peripheral venous blood sample was drawn from each subject after 12 hours of fasting. Parameters of glucose homeostasis (fasting plasma glucose, glycated hemoglobin (HBA1c), C-peptide and insulin) and lipid profile (total cholesterol, high-density lipoprotein cholesterol, low-density lipoprotein cholesterol and triacylglycerides) were assessed in a certified hospital laboratory. Serum zonulin was detected using Human Zonulin ELISA Kit (Elabscience).

Insulin sensitivity and secretion

Insulin sensitivity and secretion were evaluated using data from oral glucose tolerance test (OGGT). OGTT (75g glucose) was performed after 12-hour fasting according to WHO recommendation. First, baseline blood samples were drawn, than the sampling was done 30-minute intervals for two hours yielding 5 values for each subject. Incremental AUCs for glucose and insulin were calculated using trapezoid rule. Insulin sensitivity alone was expressed as Matsuda Index (MI) as published [5].

In detail. In AUC calculation, the fasting serum glucose level was subtracted from each value to adjust for variability in fasting serum glucose levels between subjects giving the incremental AUC. The formula used for calculation of AUC was:

$$AUC = 0 + 2 \times (G|30 - G_0) + 2 \times (G|60 - G_0) + 2 \times \frac{(G|90 - G_0) + (G_{120} - G_0)}{8} \times 120$$

where G with lower index number represents serum glucose (mmol/l) in the respective time during OGGT.

The formula used for calculation of MI was:

$$MI = \frac{10000}{\sqrt[2]{(I_0 \times G_0) \times (I_{mean} \times G_{mean})}}$$

where I_0 and G_0 stand for fasting serum insulin (mU/l) and glucose (mg/dl) and I_{mean} a G_{mean} stand for average serum insulin (mU/l) and glucose (mg/dl) during oGGT, which were counted as:

$$G_{mean} = \frac{G_0 + 2 \times G_{30} + 2 \times G_{60} + 2 \times G_{90} + G_{120}}{8}$$

$$I_{mean} = \frac{I_0 + 2 \times I_{30} + 2 \times I_{60} + 2 \times I_{90} + I_{120}}{8}$$

Sample manipulation and storage

Stool collected at home was immediately stored at −20°C until transported in the frozen state to the laboratory. Once thawed, the four fold of water was added to the sample (up to 10 g), and samples were homogenized using stomacher (BioPro, CR). Immediately after homogenization, an aliquot (600 ul) was taken for DNA analysis. pH was determined in the rest of the sample and the homogenate was sonicated for 1 minute at the maximal amplitude and cycles (UP200S, Heischler Ultrasound Technology). Sonicated samples were used for dry mass estimation and aliquoted and stored at -50 °C until metabolome analysis.

Blood samples were drawn from median cubital vein into Vacutainer tube. For serum, the blood was left standing on the bench for 30 min to clot and then separated by centrifugation. For plasma, the blood was collected into Vacutainer with the anticoagulant, immediately mixed by gently inverting the tube five times and then separated by centrifugation. Parameters of glucose homeostasis were measured in a certified hospital laboratory: fasting plasma glucose using the hexokinase reaction (KONELAB, Dreieich, Germany); C-peptide by using solid-phase competitive chemiluminescent enzyme immunoassay (Immulite 2000, Los Angeles, CA, USA); HbA1c by using high-pressure liquid boronate affinity chromatography (Primus Corporation, Kansas city, MO, USA); and insulin using solid-phase competitive chemiluminescent enzyme immunoassay (Immulite 2000). For the lipid profile, we measured total cholesterol and triglycerides using an enzymatic method kit (KONELAB); high-density lipoprotein–cholesterol measured using a polyethylene glycol-modified enzymatic assay kit (ROCHE, Basel, Switzerland); and low-density lipoprotein–cholesterol calculated using the standard Friedewald equation.

Gut microbiome analysis

Fecal Sample Collection and Bacterial DNA Extraction.

Stool collected at home was immediately stored at -20°C until transported in the frozen state to the laboratory. Until isolation, samples were stored at -50 °C. For DNA isolation, 200-250 mg of stool was cut on dry ice. DNA was isolated by QIAmp PowerFecal DNA Kit (Qiagen), according to manufacturer recommendation.

16S rRNA gene Library Preparation and Sequencing

Quality of DNA was determined using gel electrophoresis and concentration was assessed spectrophotometrically using microplate reader (Synergy Mx, BioTek, USA). For identification of bacteria presented in samples, the sequencing of 16S rRNA gene was performed. Extracted DNA was used as a template in amplicon PCR to target the hypervariable region V4 of the bacterial 16S rRNA. The library was prepared according to the Illumina 16S Metagenomic sequencing Library Preparation protocol with some deviations described below (1). The total reaction volume of PCR was 30 μl consisting of 15 μl Q5 HighFidelity 2x MM (BioLabs, New England), 1.5 μl of each 10 μM primer, 9 μl of PCR water and and 3 μl of template. The cycling parameters included initial denaturation at 98 °C for 30 s, followed by 30 cycles of 10 s denaturation at 98 °C, 15 s annealing at 55 °C and 30 s extension at 72 °C, followed by final extension at 72 °C for 2 min. The primer pair consisting of Illumina overhang nucleotide sequences, an inner tag and gene-specific sequences. The Illumina overhang served to ligate the Illumina index and adapter. Each inner tag, i.e. a unique sequence of 7–9 bp, was designed to differentiate samples into groups. The amplified PCR products were determined by gel electrophoresis. PCR clean-up was performed with SPRIselect beads (Beckman Coulter Genomics). Samples with different inner tags were equimolarly pooled based on fluorometrically measured concentration using Qubit® dsDNA HS Assay Kit (Invitrogen™, USA) and microplate reader (Synergy Mx, BioTek, USA). Pools were used as a template for a second PCR with Nextera XT indexes (Illumina, USA). Differently indexed samples were checked and quantified using the three methods: qPCR using LightCycler 480 Instrument (Roche, USA) and KAPA Library Quantification Complete Kit (Roche, USA); 2100 Bioanalyzer Instrument using the High Sensitivity D1000 ScreenTape (Agilent Technologies, USA) and microplate reader (Synergy Mx, BioTek, USA) Qubit® using dsDNA HS Assay Kit (Invitrogen™, USA). Samples were equimolarly pooled according to the measured concentration. The prepared library was checked with the same methods and concentration was measured shortly prior sequencing. The final

library was diluted to a concentration of 8 pM and 20 % of PhiX DNA (Illumina, USA) was added. Sequencing was performed with the Miseq reagent kit V2 using a MiSeq instrument according to the manufacturer's instructions (Illumina, USA).

Data processing

Paired reads from 16s rRNA sequencing were first processed using an in-house pipeline implemented in Python 3. Steps of processing included trimming of low-quality 3' ends of reads, removal of read pairs containing unspecified base N and removal of pairs containing very short reads. In order to minimize sequencing and PCR-derived error, forward and reverse reads were denoised using the DADA2 amplicon denoising R package (2). Following denoising, the forward and reverse reads were joined into a single longer read using the fastq-join read joining utility (3). In order to be joined, reads in pairs had to have an overlap of at least 20 base pairs with no mismatches allowed. Pairs in which this was not the case were discarded. As the final step, chimeric sequences were removed from the joined reads using the remove Bimera function of the DADA2 R package. Subsequent taxonomic assignment was conducted by the uclust-consensus method from the QIIME (4) microbial analysis framework using the Silva v. 123 (5) reference database. In cross-sectional study, we found 44,332 ASVs and identified 13 phyla, 30 classes, 56 orders, 104 families and 367 genera. The median sequencing coverage was 27,515 ASV per sample (min 14,382; max 74,538). In prospective study, we detected 9114 ASVs and identified 15 phyla, 37 classes, 60 orders, 97 families and 285 genera. The median sequencing coverage was 28,383 ASV per sample (min 14,382; max 55,923).

Determination of short-chain fatty acids in serum

SCFAs were analyzed in plasma by LC-MS according to a method described before (6) with minor modifications. Briefly, fifty microliters of a mixed standard solution containing 4 mM of formic acid and acetic acid, 2 mM of propionic acid, and 1 mM of each of the other six SCFAs were added to a 2 mL borosilicate test tube that contained 1 mg of ^{13}C6-3NPH HCl. Twenty-five microliters of 120 mM EDC-6% pyridine solution and twenty-five microliters 75% MeOH were then added to the mixture. The mixture was reacted at 4 °C for 4 hours. Twenty-five microliters quinic acid in MeOH was added and quenching proceeded for 45 min. After quenching, the mixture was transferred to a volumetric flask with 10% MeOH and diluted with the same solvent to 100 mL. This solution was used as the internal standard mix and was stored in aliquots at -20 °C. In total, 10 µl plasma was incubated with 60 µl 75% methanol, 10 µl 200 mM 3-NPH and 10 µl 120 mM EDC-6% pyridine at ambient temperature for 45 min with shaking. The reaction was quenched by addition of 10 µl of 200 mM quinic acid (15 min with shaking). The samples were centrifuged at 15 000 g for 5 min and the supernatant moved to a new tube. The samples were made up to 1 mL by 10% methanol in water and again centrifuged at 15 000 g for 5 min. In total, 100 µl of the derivatised (^{12}C 3NPH) sample was mixed with 100 µl of labelled (^{13}C 3NPH) internal standard. A mixed external standard solution containing 3,2 µM – 0,63 nM of formic acid and acetic acid, 3,2 µM – 0,31 nM of propionic acid, and 0,8 µM – 0,16 nM of each of the other six SCFAs were always prepared fresh and used for each batch. Samples were analyzed by a 6500+ QTRAP triple-quadrupole mass spectrometer (AB Sciex, 11432 Stockholm, Sweden) which was equipped with an APCI source and operated in the negative-ion mode. Chromatographic separations were performed on a Phenomenex Kinetix Core-Shell C18 (2.1, 100 mm, 1.7 um 100 Å) UPLC column with SecurityGuard ULTRA Cartridges (C18 2.1mm ID) (changed at regular intervals at). The column was backflushed for 60 min between each batch to ensure good chromatographic separation. Water (100% solvent A) and acetonitrile (100% solvent B) was the mobile phases for gradient elution. The column flow rate was 0.4 mL/min and the column temperature was 40 °C, the autosampler was kept at 4 °C. LC starting conditions at 0.5% B, held for 3 min, 3 min 2.5% B ramping linearly to 17% B at 6 min, then to 45% B at 10 min and 55% B at 13 min. Followed by a flush (100% B) and recondition (0.5% B), total runtime 15 min. The MRM transitions were optimized for the analytes one by one by direct infusion of the derivatives containing 10 µM of each fatty acid, essentially as according to Han et al. (7). The Q1/Q3 pairs were used in the MRM scan mode to optimize the collision energies for each analyte, and the two most sensitive pairs per analyte were used for the subsequent analyses. The retention time window for the scheduled MRM was 1 min for each analyte. The two MRM

transitions per analyte, the Q1/Q3 pair that showed the higher sensitivity was selected as the MRM transition for quantitation. The other transition acted as a qualifier for the purpose of verification of the identity of the molecule. UPLC/MRM-MS data was acquired in the "scheduled MRM" mode using the Analyst 1.5 software and data processing was performed using the MultiQuant 3.0.3 software (AB Sciex, 11432 Stockholm, Sweden).
Standards for SCFAs used were: Formic Acid (C1) (Scharlau), acetic acid (C2) (Honeywell), propionic acid (C3) (Alfa Aesar), butyric acid (C4) (Sigma Aldrich), isobutyric acid (C4) (Alfa Aesar), Succinic acid (C4) (Acros), isovaleric acid (C5) (Sigma Aldrich), valeric acid (C5) (Alfa Aesar) and caproic acid (C6) (Sigma Aldrich). Analytical reagent-grade 3-nitrophenylhydrazine (3NPH)-HCl (97%), 2-nitrophenylhydrazine N-(3-dimethylaminopropyl)-N0-ethylcarbodiimide (EDC) HCl, quinic acid, HPLC grade pyridine and Lichrosol reagent grade MeOH and water was obtained from Sigma–Aldrich. Acetonitrile Optima LCMS Grade was obtained from Fisher scientific. ^{13}C6-3NPH-HCl was custom synthesized to us by IsoSciences Inc. (King of Prussia, PA, USA) (catalogue 13309). This custom-synthesized compound was structurally confirmed by 1H NMR spectroscopy and by MS/MS on a triple-quadruple mass spectrometer.

Volatile compounds analysis of feces

Stool was homogenized and diluted to equivalent of 1% (*w/w*) dry mass. This was pippeted into a 10 mL vial for headspace analysis, and prior sealing with a magnetic cap, 20 µl of sodium azide water solution (0.2%, *w/v*) was added as a bacteriostatic agent. Volatiles fingerprinting was performed using an Agilent 7890B gas chromatograph coupled to Leco Pegasus 4D time of flight mass spectrometer. The instrument was equipped with a multi-purpose autosampler (MPS, Gerstel, USA), performing heated incubation, steering, and volatiles collection onto a solid-phase microextraction fiber with a divinylbenzen/carboxen/ polydimethylsiloxan (DVB/CAR/PDMS 50/30 µm) coating from Supelco (USA).

The sample was incubated for 10 min and volatiles extracted onto a fibers stationary phase for 20 minutes, both at steering at temperature of 60 °C. Separation was performed on GC capillary column HP-Innowax (30 m × 0.25 mm i.d., 0.25 µm film thickness; Agilent Technologies, USA) with splitless injection at 265 °C. The GC oven temperature program was as follows: 40 °C for 1 min; then ramped at a rate of 10 °C/min to 180 °C; then at 20 °C/min to 260 °C and held for 6 min for a total GC run time of 25 min.

Time of flight mass spectrometer was operated with acquisition speed of 10 Hz to obtain full spectral information in a mass range 35–350 Da. Peak find,mass spectral deconvolution and subsequent peak alignment were performed in ChromaTOF software (LECO, USA). Compounds with a quantification mass signal to noise ratio (S/N), higher than 100 and present in more than 50 % of smallest sample class, were selected for alignment. For signals from different samples, to be listed in the aligned table as a single compound, retention time (maximal difference of 5 s) and spectral similarity at least 60 % must be met. n the aligned table, areas of quantification masses for each aligned compound, with tentative identificationwere provided. This tentative ID is based on spectral similarity of deconvoluted mass spectrum of signal and spectra in NIST 2017 mass spectral library. Further confirmation of signals identity was based on comparison of measured retention index and retention indexes in the NIST library. An aligned table was exported to Microsoft Excel, where constant sum normalization was performed. Thus each compounds quantification mass area was divided by sum of all signals quantification mass areas in respective sample.

NMR analysis

Serum samples were analyzed after protein precipitation. Aliquot of 220 µl serum sample was mixed with 440 µl cold methanol. The mixture was kept in freezer at -20 °C for 30 minutes and then centrifuged at 18 620 g for 10 minutes at 4 °C. The supernatant was transferred into fresh vial and vacuum dried. Evaporated supernatant was dissolved in 450 µl D_2O with 50 µl 1.5 M phosphate buffer and 50 µl 0.1% TSP, and then transferred into 5mm NMR tube.

NMR data were acquired on a 600 MHz Bruker Avance III spectrometer (Bruker BioSpin, Rheinstetten, Germany) equipped with a 5mm TCI cryogenic probe head. All experiments were performed using Topspin 3.5 software at 300 K with automatic tuning and matching, shimming and adjusting 90° pulse

length for each sample. Serum data were analyzed from Carr-Purcell-Meiboom-Gill (CPMG) spectra acquired by cpmgpr1d pulse sequence with following acquisition parameters; number of scans NS=192, spectral width SW=20 ppm, 64k of data points (TD), relaxation delay for water presaturation d1=4 s, echo time 0.3 ms, loop for T2 filter 126. *J*-resolved experiment (NS=2, SW=16, TD=8k, number of increments=40, SW=78.125 Hz in the indirect dimension, d1=2 s) was performed on each sample to facilitate metabolite identification. Additional heteronuclear single quantum correlation (HSQC) and total correlation spectroscopy (TOCSY) experiments were executed for selected samples.

Acquired data were processed with Topspin 3.5 software. CPMG spectra were line broadened (0.3 Hz), automatically phased, baseline corrected and referenced to the signal of TSP. The regions with signal of water and methanol were excluded and then spectra were normalized using probabilistic quotient normalization (PQN) method (8) to the pooled lean healthy group. Individual metabolites were identified using Chenomx software (Chenomx Inc., Edmonton, AB, Canada) and their proton and carbon data were then compared with the HMDB database (9). Metabolite concentrations were expressed as normalized intensities of corresponding signals in CPMG spectra.

Data analysis

Identification of discriminating features between cohorts

The statistical analyses were performed using R software packages and in-house scripts (21). For individual tasks, the following R packages were used: composition (centered log-ratio transformation), zCompositions (zero multiplicative replacement) vegan (PERMANOVA), phyloseq (α-diversity), effsize (Cliff's delta), glmnet (LASSO logistic regression). Clinical characteristics of the observational sample were compared using standard tests. Prior to further analyses, all variables for which the sum of counts was below 0.01% of the total sum of all counts were removed from microbiome data and all variables for which the sum of AUC was below 0.01% of the total AUC were removed from fecal metabolome data. The microbiome and VOCs data were treated as compositional (proportions of total read count in each sample or proportion of the total area of selected masses), and before all statistical analyses, the data were transformed by centered log-ratio (clr) transformation with a multiplicative simple replacement for handling zero values. All data were scaled (z-score) before applying PERMANOVA, principal component analysis (PCA), or LASSO regression PCA was used to investigate possible sample clustering in each dataset. For each data type, multivariable statistics (PERMANOVA, 10000 permutations) were applied to test for differences between the groups. Univariable statistical analyses were performed by the Mann-Whitney-Wilcoxon test when testing two groups and the Kruskal-Wallis test when testing at least three groups. The results were adjusted for multiple hypothesis testing by the Benjamini-Hochberg procedure with cut-off levels at a false discovery rate equal to 0.10. We analyzed the discriminatory power of each omics dataset using machine learning; specifically, we used logistic regression with L1 penalization (i.e., LASSO) with ten times repeated 10-fold cross-validation. To handle imbalanced groups, each sample was assigned a weight inversely proportional to the number of samples within the respective group. The validity of a model was verified using a permutation test with 50 repetitions. Correlation networks based on Spearman's correlation coefficient were used to assess the correlation between the studied variables.

Classification into patients groups using machine learning

To classify patients into groups, we employed machine learning approach called Logistic regression with L1 penalization (i.e., Lasso) with 10 times repeated 10-fold cross-validation. To handle imbalanced groups, each sample have a weight inversely proportional to the number of samples within that group. The validity of each model was verified using permutation test with 50 repetitions. Lambda parameter was obtained by cross-validation using glmnet package and the miss-classification error was used as a loss function. All the data were scaled (z-score) before learning the models.

Network analysis: Lasso and univariate differential abundance analysis

For each model obtained using Lasso, we created a correlation network based on Spearman correlation coefficient between discriminating variables selected by univariate analysis and Lasso model. Only strong correlations are shown:

- microbiome, genus: $|\rho|>=0.3$
- VOC: $|\rho|>=0.4$
- serum metabolome: $|\rho|>=0.3$
- all variables: $|\rho|>=0.4$

Predictive signatures in inulin interventions

The effect of each possible predictor variable on the clinical outcomes was assessed by linear regression

$$V_{p,y}^{(B)} - V_{p,y}^{(A)} = \beta_0 + \beta_y V_{p,y}^{(A)} + \beta_x z(V_{p,x}^{(A)}) + \epsilon_{p,x,y}$$

where: *x,* name the predictor variable; *y*, name of the outcome variable; $V_{p,x}^{(A)}$, value of a predictor *x* of a patient *p* in time *A*; $V_{p,x}^{(B)}$, value of a predictor *x* of a patient *p* in time *B*; $V_{p,y}^{(A)}$, value of an outcome *y* of a patient *p* in time *A;* β_x, β_y coefficients given by the model ; z(), z-score standardization function; $\epsilon_{p,x,y}$ error term.

That is, we fit a model to predict the difference in the clinical outcome after intervention using individual predictors before intervention, confounding on the clinical value before intervention. Along with individual p-values for the variables, we report the resampled R^2 obtained using bootstrapping (50 iterations). We omitted variables with significant coefficients having high leverage observations. Such variables were identified by analyzing the graph of the univariable linear regression.

Supplemental references

1. Klindworth, A. *et al*. Evaluation of general 16S ribosomal RNA gene PCR primers for classical and next-generation sequencing-based diversity studies. Nucleic Acids Res*.* 41, e1 (2013).
2. Callahan B.J., McMurdie P.J., Rosen M.J., Han A.W., Johnson A.J.A., Holmes S.P. DADA2: High-resolution sample inference from Illumina amplicon data, Nat Methods 13, 581–583 (2016).
3. Aronesty,E. Comparison of sequencing utility programs. Open Bioinform. J., 7, 1–8 (2013).
4. Caporaso J., Kuczynski J., Stombaugh J., Bittinger K., ET AL. QIIME allows analysis of high-throughput community sequencing data, Nat Methods 7, 335–336 (2010).
5. Quast, C. et al. The SILVA ribosomal RNA gene database project: improved data processing and web-based tools. Nucleic Acids Res. 41, D590–D596 (2013).
6. Han, J., Lin, K., Sequeira, C. & Borchers, C.H. An isotope-labeled chemical derivatization method for the quantitation of short-chain fatty acids in human feces by liquid chromatography–tandem mass spectrometry. *Analytica chimica acta* **854**, 86-94 (2015).
7. Dieterle F., Ross A., Schlotterbeck G., Senn H., Probabilistic quotient normalization as robust method to account for dilution of complex biological mixtures. Application in 1H NMR metabonomics. Anal Chem. 78(13), 4281-4290 (2006)

8. Wishart D.S. *et al*. HMDB 4.0: the human metabolome database for 2018. Nucleic Acids Res. 46(D1), D608-D617 (2018)
9. Kuhn, M. Caret package. Journal of Statistical Software 28(5), (2008)

2 Supplementary Figures and Tables

Table S1 *List of quantified metabolites in serum and fecal extracts using NMR (with corresponding ^{1}H and ^{13}C chemical shifts).*

	**metabolite**	**^{1}H chemical shift [ppm]**	**^{13}C chemical shift [ppm]**
1.	Lipoproteins CH_3-	**0.80 – 0.87**	16.8
3.	3-Methyl-2-oxovalerate	0.89 (t), **1.10** (d)	13.3, 16.4
5.	2-Hydroxybutyrate	**0.90** (t), 1.66 (m), 4.00 (m)	11.4
6.	2-Oxoisocaproate	**0.94** (d), 2.61 (d)	24.5, 51.0
7.	Isoleucine	0.94 (t), **1.01** (d), 1.27 (m), 1.47 (m), 3.68 (d)	13.9, 17.4, 27.2, 38.6, 62.3
8.	Leucine	**0.96** (d), 0.97 (d), 1.71 (m), 1.72 (m), 3.73 (m)	23.7, 24.8, 26.9, 42.6, 56.2
9.	Valine	0.99 (d), **1.05** (d), 2.27 (m), 3.62 (d)	19.4, 20.7, 31.8, 63.1
11.	3-Hydroxyisobutyrate	**1.07** (d), 2.49 (m), 3.54 (m), 3.70 (m)	16.8
12.	2-Oxoisovalerate	**1.12** (d)	19.1
13.	Ethanol	1.19 (t), **3.65** (q)	n.d.
14.	3-Hydroxybutyrate	**1.20** (d), 2.31 (dd), 2.40 (dd)	24.5, 68.4
16.	Lactate	1.33 (d), **4.11** (q)	22.9, 71.2
17.	Threonine	1.33 (d), 3.59 (d), **4.25** (m)	22.3, 63.2, 68.9
19.	Lysine	1.45 (m), 1.52 (m), 1.73 (m), 1.91 (m), **3.04** (t)	24.2, 29.1, 32.7, 41.8
20.	Alanine	**1.49** (d), 3.79 (q)	18.9, 53.3
21.	Acetate	**1.92** (s)	26.1
22.	Ornithine	1.95 (m), **3.06** (t)	30.3, 41.5
23.	Proline	2.01 (dd), 2.08 (m), 2.36 (m), 3.43 (m), **4.14** (m)	26.5, 31.8, 48.8, 63.9
25.	Glutamine	2.14 (m), **2.46** (m)	29.1, 33.6

27.	Acetone	**2.23** (s)	n.d.
29.	Pyruvate	**2.38** (s)	n.d.
31.	Citrate	2.55 (d), **2.69** (d)	48.5
33.	Dimethylamine	**2.72** (s)	37.4
34.	Asparagine	**2.87** (dd), 2.95 (dd), 4.00 (m)	37.5, 54.0
37.	Dimethylsulfone[x]	**3.16** (s)	n.d.
40.	Tyrosine	3.06 (dd), 3.21 (dd), 3.95 (dd), **6.91** (m), 7.20 (m)	38.3, 58.8, 118.6, 133.5
41.	Phenylalanine	3.13 (dd), 3.29 (dd), 4.00 (m), 7.34 (m), 7.38 (m), **7.44** (m)	39.1, 58.8, 130.4, 131.8, 132.2
42.	Glycine	**3.57** (s)	44.2
44.	Histidine	3.99 (dd), **7.10** (s), 7.90 (s)	57.4, 119.8, 138.9
45.	Tryptophan	4.06 (dd), 7.21 (m), 7.29 (m), 7.33 (m), 7.55 (m), **7.74** (m)	29.2, 57.8, 114.7, 121.2, 122.2, 124.8, 127.9
49.	Glucose	4.66 (d), **5.25** (d), 3.26 (dd), 3.41 (m), 3.37 (m), 3.50 (dd), 3.55 (dd), 3.72 (m), 3.77 (dd), 3.84 (m), 3.90 (dd)	94.8, 98.663.3, 63.5, 72.3, 74.2, 75.5, 76.9, 78.5, 78.7
50.	Mannose	4.90 (d), **5.18** (d)	96.4, 96.7
55.	Formate	**8.46**	n.d.
58.	Glycerol	3.56 (dd), **3.66** (dd)	65.3
59.	Unidentified N-acetyl	**2.07** (s)	24.9
63.	Unidentified	**1.43** (d)	n.d.

The table lists all metabolites quantified in serum; the signals used for metabolite quantification are in bold. Signal multiplicity is marked as follows: (s)-singlet, (d)-doublet, (t)-triplet, (dd)-doublet of doublets, (q)-quartet, (m)-multiplet; n.d. – signals not detected; [x]-tentative assignment.

Table S2 *Gut microbiome composition: phylum level.*

	p	FDR	Dunn´s			Cliff´s			LH			OB			DM		
			OB - LH	DM - LH	DM - OB	OB - LH	DM - LH	DM - OB	Q1	median	Q3	Q1	median	Q3	Q1	median	Q3
Firmicutes	0.039	0.195	0.395	0.306	0.034	-0.14	0.13	0.28	42.7	50.1	55.4	33.7	45.2	56.7	40.8	48.8	61.2
Bacteroidetes	0.986	0.986	>0.999	0.967	>0.999	0.00	-0.01	0.03	33.3	40.6	45.4	27.0	34.8	52.4	23.2	34.2	47.7
Actinobacteria	0.011	0.135	0.016	0.012	0.985	-0.36	-0.35	-0.01	1.52	4.2	6.9	0.9	1.9	3.7	0.9	1.6	3.1
Proteobacteria	0.473	0.710	0.584	0.703	0.622	0.07	0.16	0.09	0.80	1.5	3.5	0.8	1.7	3.5	0.8	1.9	4.2
Verrucomicrobia	0.865	0.927	>0.999	>0.999	0.861	0.05	0.07	0.02	0.00	0.4	2.2	0.0	0.3	3.6	0.0	0.1	8.2
Tenericutes	0.099	0.297	0.247	0.099	0.310	-0.15	-0.28	-0.14	0.00	0.0	0.8	0.0	0.0	0.6	0.0	0.0	0.1
Cyanobacteria	0.766	0.927	>0.999	0.826	0.929	0.09	0.08	-0.01	0.00	0.0	0.1	0.0	0.0	0.1	0.0	0.0	0.1
Fusobacteria	0.018	0.135	0.793	0.030	0.034	0.04	0.31	0.28	0.00	0.0	0.0	0.0	0.0	0.0	0.0	0.0	0.0
Lentisphaerae	0.058	0.218	0.073	0.058	0.937	-0.28	-0.29	0.02	0.00	0.0	0.0	0.0	0.0	0.0	0.0	0.0	0.0
Saccharibacteria	0.170	0.425	0.412	0.205	0.305	0.10	0.25	0.14	0.00	0.0	0.0	0.0	0.0	0.0	0.0	0.0	0.0
Elusimicrobia	0.305	0.572	0.929	0.394	0.429	-0.01	0.14	0.17	0.00	0.0	0.0	0.0	0.0	0.0	0.0	0.0	0.0
Euryarchaeota	0.405	0.675	0.501	0.926	0.661	0.13	-0.01	-0.14	0.00	0.0	0.0	0.0	0.0	0.1	0.0	0.0	0.0
Synergistetes	0.835	0.927	0.882	>0.999	>0.999	-0.02	0.04	0.07	0.00	0.0	0.0	0.0	0.0	0.0	0.0	0.0	0.0

Table S3 *Gut microbiome composition: genus level.*

	p	FDR	core	Butyrate producer	Dunn´s			Cliff´s			LH			OB			DM		
					OB-LH	DM-LH	DM-OB	OB-LH	DM-LH	DM-OB	Q1	median	Q3	Q1	median	Q3	Q1	median	Q3
Faecalibacterium	0.022	0.096	YES	YES	0.032	0.021	0.964	-0.34	-0.32	-0.01	2.10	3.02	4.56	1.05	1.66	2.73	0.75	1.22	4.44
Bifidobacterium	0.016	0.089	YES	NO	0.014	0.036	0.617	-0.37	-0.30	0.05	0.55	1.93	4.63	0.16	0.49	1.47	0.12	0.54	1.11
Blautia	<0.001	<0.001	YES	POTENTIAL	<0.001	0.028	0.033	-0.51	-0.35	0.28	1.15	1.82	2.25	0.24	0.63	1.04	0.36	0.69	1.05
Lachnospiraceae_ Unassigned	0.003	0.033	YES	POTENTIAL	0.002	0.106	0.087	-0.42	-0.23	0.22	1.13	1.72	2.84	0.47	0.80	1.40	0.60	0.90	1.55
Christensenellaceae R-7 group	0.003	0.033	YES	YES	0.300	0.005	0.012	-0.15	-0.41	-0.31	0.20	1.24	2.20	0.12	0.75	1.84	0.01	0.20	0.80
Lachnospiraceae NK4A136 group	0.024	0.096	YES	YES	0.771	0.035	0.047	-0.04	-0.30	-0.27	0.21	0.71	1.78	0.16	0.64	1.35	0.03	0.21	0.86
Fusicatenibacter	<0.001	<0.001	YES	NO	<0.001	0.002	0.418	-0.53	-0.44	0.09	0.33	0.64	1.05	0.06	0.24	0.43	0.11	0.20	0.34
Dorea	0.021	0.096	YES	NO	0.017	0.196	0.180	-0.36	-0.20	0.15	0.36	0.58	0.71	0.14	0.25	0.47	0.18	0.29	0.48
Anaerostipes	0.001	0.021	YES	YES	<0.001	0.026	0.130	-0.49	-0.33	0.17	0.19	0.42	1.01	0.05	0.12	0.22	0.07	0.13	0.29
[Eubacterium] hallii group	0.001	0.021	YES	YES	0.001	0.004	0.523	-0.48	-0.40	0.07	0.18	0.39	0.63	0.05	0.10	0.20	0.03	0.10	0.22
Lachnospiraceae Incertae Sedis	0.020	0.096	YES	POTENTIAL	0.036	0.020	0.490	-0.29	-0.37	-0.07	0.10	0.31	0.49	0.00	0.07	0.54	0.00	0.03	0.30
Erysipelotrichaceae UCG-003	0.002	0.029	YES	NO	0.002	0.010	0.526	-0.45	-0.35	0.06	0.16	0.23	0.56	0.00	0.07	0.27	0.00	0.08	0.25
Lachnospiraceae ND3007 group	0.024	0.096	YES	YES	0.021	0.038	0.844	-0.34	-0.31	-0.04	0.05	0.17	0.24	0.02	0.05	0.14	0.00	0.04	0.11
Pseudobutyrivibrio	0.016	0.089	YES	POTENTIAL	0.354	0.209	0.012	-0.12	0.20	0.32	0.31	0.68	2.23	0.10	0.58	1.33	0.36	1.03	2.16

Lachnoclostridium	0.014	0.089	YES	POTEN-TIAL	0.492	0.026	0.025	0.08	0.36	0.26	0.88	1.71	2.20	0.72	1.45	3.26	1.34	2.50	4.72
Lachnospiraceae FCS020 group	<0.001	<0.001	NO	NO	<0.001	0.002	0.466	-0.49	-0.45	0.09	0.01	0.04	0.09	0.00	0.00	0.02	0.00	0.00	0.02
Lachnospiraceae UCG-008	0.006	0.054	NO	NO	0.005	0.015	0.628	-0.41	-0.34	0.05	0.02	0.04	0.06	0.00	0.01	0.03	0.00	0.01	0.03
[Ruminococcus] gauvreauii group	0.023	0.096	NO	NO	0.029	0.026	0.928	-0.35	-0.30	0.00	0.01	0.11	0.21	0.00	0.03	0.08	0.00	0.01	0.06
Family XIII AD3011 group	0.016	0.089	NO	NO	0.528	0.121	0.013	0.08	-0.24	-0.32	0.02	0.02	0.07	0.01	0.04	0.08	0.00	0.01	0.05
Marvinbryantia	0.003	0.033	NO	YES	0.003	0.134	0.070	-0.41	-0.22	0.23	0.00	0.00	0.04	0.00	0.00	0.00	0.00	0.00	0.00
Tyzzerella 3	0.004	0.041	NO	NO	0.975	0.017	0.006	-0.01	0.34	0.35	0.00	0.00	0.00	0.00	0.00	0.00	0.00	0.00	0.06
Bacteroidales_un-assigned	0.021	0.096	NO	NO	0.020	0.032	0.801	0.30	0.37	0.02	0.00	0.00	0.00	0.00	0.00	0.00	0.00	0.00	0.00
Flavonifractor	0.023	0.096	NO	YES	0.126	0.019	0.180	0.20	0.37	0.17	0.00	0.00	0.06	0.00	0.04	0.17	0.00	0.07	0.28
Prevotella 7	0.016	0.089	NO	NO	0.019	0.021	0.863	0.32	0.37	-0.04	0.00	0.00	0.00	0.00	0.00	0.14	0.00	0.00	0.00
Megamonas	0.006	0.054	NO	NO	0.733	0.011	0.013	0.03	0.38	0.31	0.00	0.00	0.00	0.00	0.00	0.00	0.00	0.00	0.06
Mitsuokella	0.025	0.097	NO	POTEN-TIAL	0.134	0.020	0.180	0.20	0.39	0.14	0.00	0.00	0.00	0.00	0.00	0.00	0.00	0.00	0.00
Prevotellaceae _uncultured	0.014	0.089	NO	NO	0.014	0.021	0.777	0.32	0.39	0.02	0.00	0.00	0.00	0.00	0.00	0.00	0.00	0.00	0.00
Ruminococcaceae UCG-004	0.024	0.096	NO	NO	0.042	0.024	0.489	0.26	0.39	0.06	0.00	0.00	0.00	0.00	0.00	0.05	0.00	0.00	0.00
Slackia	0.024	0.096	NO	NO	0.170	0.019	0.137	0.17	0.41	0.15	0.00	0.00	0.07	0.00	0.00	0.24	0.00	0.05	0.15
Catenibacterium	0.011	0.083	NO	POTEN-TIAL	0.075	0.008	0.162	0.25	0.41	0.15	0.00	0.00	0.00	0.00	0.00	0.01	0.00	0.00	0.05
Succinivibrio	0.015	0.089	NO	NO	0.151	0.012	0.107	0.19	0.41	0.17	0.00	0.00	0.00	0.00	0.00	0.00	0.00	0.00	0.00
Fusobacterium	0.002	0.029	NO	POTEN-TIAL	0.250	0.003	0.012	0.14	0.45	0.29	0.00	0.00	0.00	0.00	0.00	0.00	0.00	0.00	0.00
Alloprevotella	0.008	0.064	NO	NO	0.022	0.007	0.366	0.29	0.45	0.09	0.00	0.00	0.00	0.00	0.00	0.00	0.00	0.00	0.00
Tyzzerella 4	0.007	0.059	NO	NO	0.069	0.005	0.127	0.24	0.45	0.16	0.00	0.00	0.00	0.00	0.00	0.00	0.00	0.00	0.00
Megasphaera	0.002	0.029	NO	YES	0.028	0.001	0.122	0.29	0.49	0.17	0.00	0.00	0.00	0.00	0.00	0.62	0.00	0.00	2.98
Desulfovibrio	<0.001	<0.001	NO	NO	0.001	<0.001	0.428	0.44	0.54	0.09	0.00	0.00	0.02	0.00	0.05	0.38	0.00	0.08	0.47

Table S4 *Gut metabolome composition: VOCs.*

	p-value	FDR	Dunn´s			Cliff´s			LH			OB			DM		
			OB-LH	DM-LH	DM-OB	OB-LH	DM-LH	DM-OB	Q1	median	Q3	Q1	median	Q3	Q1	median	Q3
propyl acetate	<0.001	<0.001	<0.001	0.001	0.575	-0.52	-0.48	0.07	0.09	0.23	0.57	0.02	0.07	0.13	0.04	0.09	0.16
nonanoic acid	<0.001	<0.001	<0.001	<0.001	0.570	0.66	0.64	-0.08	0.00	0.03	0.06	0.19	0.49	0.70	0.20	0.39	0.68
decane	0.002	0.048	0.036	0.001	0.076	-0.29	-0.51	-0.21	0.12	0.21	0.38	0.00	0.14	0.26	0.00	0.07	0.19
tetradecanal	0.003	0.054	0.112	0.002	0.035	-0.22	-0.45	-0.28	0.05	0.12	0.40	0.05	0.11	0.18	0.03	0.05	0.11
humulene	0.007	0.094	0.143	0.006	0.060	-0.19	-0.44	-0.24	0.06	0.13	0.42	0.04	0.15	0.30	0.01	0.08	0.15
trans-ocimene	0.009	0.094	0.179	0.008	0.058	-0.20	-0.39	-0.26	0.11	0.24	1.61	0.12	0.22	0.48	0.08	0.15	0.36
2-octanol	0.010	0.094	0.068	0.007	0.159	-0.26	-0.43	-0.17	0.10	0.13	0.26	0.05	0.08	0.14	0.06	0.08	0.10
benzeneacetaldehyde	0.013	0.094	0.122	0.010	0.112	-0.20	-0.41	-0.21	0.53	1.27	1.87	0.39	0.66	1.18	0.22	0.43	0.97
methyl valerate	0.013	0.094	0.198	0.286	0.011	-0.17	0.12	0.36	0.06	0.19	0.74	0.06	0.14	0.38	0.19	0.36	0.87

Only compounds meeting condition $AUC_{x,p}$ => 0.1% $AUC_{total,p}$ are shown. p, sample id.

Table S5 Serum metabolome composition

		p	FDR	Dunn´s			Cliff´s			LH			OB			DM		
				OB-LH	DM-LH	DM-OB	OB-LH	DM-LH	DM-OB	Q1	median	Q3	Q1	median	Q3	Q1	median	Q3
glucose	sacharide metabolism	<0.001	<0.001	0.012	<0.001	<0.001	0.36	0.74	0.53	2.24	2.33	2.38	2.29	2.42	2.54	2.48	2.60	2.77
mannose		<0.001	<0.001	0.006	<0.001	0.022	0.38	0.58	0.27	0.05	0.05	0.05	0.05	0.05	0.06	0.05	0.06	0.07
lactate		<0.001	<0.001	0.011	<0.001	0.049	0.33	0.55	0.22	0.69	0.77	0.95	0.80	0.96	1.06	0.92	0.99	1.22
pyruvate		0.023	0.053	0.086	0.018	0.255	0.24	0.36	0.13	0.14	0.15	0.17	0.15	0.17	0.20	0.15	0.17	0.22
tyrosine	amino acids	<0.001	<0.001	0.002	<0.001	0.220	0.41	0.54	0.14	0.14	0.16	0.18	0.16	0.18	0.20	0.18	0.19	0.21
alanine		<0.001	<0.001	0.014	<0.001	0.043	0.33	0.53	0.23	1.30	1.46	1.74	1.46	1.62	1.93	1.61	1.80	1.98
glycine		<0.001	<0.001	0.608	0.002	<0.001	0.08	-0.45	-0.48	0.44	0.49	0.57	0.42	0.51	0.64	0.34	0.39	0.47
glutamine		<0.001	<0.001	0.010	<0.001	0.030	-0.34	-0.57	-0.25	2.01	2.11	2.21	1.85	1.99	2.09	1.82	1.91	2.00
asparagine		<0.001	<0.001	<0.001	<0.001	0.054	-0.53	-0.64	-0.24	0.04	0.05	0.06	0.04	0.04	0.04	0.03	0.04	0.04
histidine		<0.001	<0.001	<0.001	<0.001	0.516	-0.64	-0.69	-0.08	0.11	0.12	0.14	0.09	0.10	0.11	0.09	0.10	0.11
2-oxoisovalerate	BCAA	0.019	0.046	0.754	0.069	0.020	-0.03	0.25	0.31	0.08	0.09	0.10	0.08	0.09	0.10	0.08	0.10	0.11
3-methyl-2-oxovalerate		0.035	0.075	0.228	0.427	0.034	-0.18	0.11	0.28	0.06	0.07	0.09	0.06	0.07	0.08	0.06	0.08	0.09
formic acid	SCFA	0.036	0.075	0.942	0.072	0.047	-0.01	0.26	0.27	0.02	0.02	0.02	0.02	0.02	0.02	0.02	0.02	0.02
succinic acid		0.006	0.019	0.006	0.011	0.803	0.37	0.36	0.04	3.05	3.39	4.06	3.32	4.13	4.87	3.37	4.28	5.94
propionic acid		0.004	0.014	0.003	0.023	0.418	0.40	0.33	-0.10	0.23	0.33	0.82	0.50	0.82	1.39	0.33	0.74	1.43
valeric acid		0.018	0.046	0.019	0.029	0.783	-0.35	-0.30	0.03	0.03	0.05	0.11	0.01	0.04	0.06	0.02	0.03	0.06
acetone	NAD+ regeneration	0.003	0.011	0.369	0.069	0.002	0.13	-0.29	-0.36	0.04	0.05	0.07	0.04	0.06	0.06	0.03	0.04	0.06
2-propanol		<0.001	<0.001	0.089	0.061	<0.001	-0.25	0.33	0.45	0.03	0.04	0.04	0.03	0.03	0.04	0.03	0.06	0.07
2-OH-butyrate		0.008	0.023	0.093	0.440	0.008	-0.24	0.11	0.33	0.14	0.17	0.19	0.13	0.16	0.18	0.15	0.17	0.20
glycerol	lipid metab.	<0.001	<0.001	<0.001	<0.001	0.919	0.51	0.49	0.02	0.08	0.09	0.11	0.10	0.12	0.15	0.10	0.12	0.16
ethanol		0.014	0.038	0.011	0.210	0.144	-0.37	-0.18	0.16	0.10	0.11	0.12	0.09	0.10	0.11	0.09	0.11	0.11

Table S6 *Effect of inulin treatment on gut microbiome composition: phylum level.*

	p	FDR	Cliff´s delta	A			B		
				Q1	median	Q3	Q1	median	Q3
Firmicutes	0.170	0.358	0.33	39.20	46.90	55.10	42.00	56.60	74.10
Bacteroidetes	0.002	0.014	-0.70	23.20	34.00	46.20	7.70	16.80	27.30
Proteobacteria	0.008	0.035	-0.62	1.10	1.90	4.20	0.40	0.70	1.80
Actinobacteria	<0.001	0.006	0.76	0.80	1.60	4.30	1.90	5.80	11.90
Verrucomicrobia	0.011	0.038	0.59	0.00	0.40	8.20	0.00	0.60	7.30
Cyanobacteria	0.445	0.606	0.19	0.00	0.01	0.1	0.02	0.04	0.06
Saccharibacteria	0.033	0.093	0.51	0.00	0.00	0.00	0.00	0.00	0.01
Chlorobi	0.160	0.358	0.34	0.00	0.00	0.00	0.00	0.00	0.00
Tenericutes	0.393	0.606	0.21	0.00	0.00	0.2	0.00	0.00	0.10
Planctomycetes	0.427	0.606	0.20	0.00	0.00	0.00	0.00	0.00	0.00
Lentisphaerae	0.463	0.606	0.18	0.00	0.00	0.00	0.00	0.00	0.00
Elusimicrobia	0.540	0.656	0.15	0.00	0.00	0.00	0.00	0.00	0.00
Euryarchaeota	0.754	0.855	0.08	0.00	0.00	0.00	0.00	0.00	0.03
Synergistetes	0.823	0.870	-0.06	0.00	0.00	0.00	0.00	0.00	0.00
Fusobacteria	0.870	0.870	-0.04	0.00	0.00	0.00	0.00	0.00	0.00

Table S7 *Effect of inulin treatment on gut microbiome composition: genus level.*

	core	butyrate producer	p	FDR	Cliff´s delta	A			B		
						Q1	median	Q3	Q1	median	Q3
increased											
Anaerostipes	YES	YES	<0.001	<0.001	0.96	0.07	0.11	0.31	0.40	0.84	1.44
[Eubacterium] hallii group	YES	YES	<0.001	<0.001	0.93	0.06	0.10	0.24	0.24	0.51	0.82
Lactobacillus	YES	NO	<0.001	0.002	0.88	0.00	0.00	0.00	0.00	0.01	0.12
Lachnospiraceae ND3007 group	YES	YES	<0.001	0.006	0.78	0.02	0.04	0.12	0.08	0.13	0.20
Fusicatenibacter	YES	NO	<0.001	0.009	0.75	0.14	0.25	0.44	0.17	0.53	1.03
Bifidobacterium	YES	NO	0.001	0.009	0.74	0.20	0.60	2.52	0.76	2.84	10.04
Blautia	YES	POTENTIAL	0.001	0.009	0.74	0.50	1.00	1.21	0.76	1.41	2.31
Lachnospiraceae_unassigned	YES	POTENTIAL	0.002	0.016	0.70	0.70	0.90	1.43	0.85	1.28	2.07
Faecalibacterium	YES	YES	0.004	0.025	0.66	0.60	1.00	3.46	1.20	2.70	4.17
Akkermansia	YES	POTENTIAL	0.007	0.041	0.62	0.00	0.42	8.16	0.03	0.57	7.30
Dorea	YES	NO	0.018	0.085	0.56	0.27	0.35	0.58	0.34	0.50	0.62
Collinsella	YES	YES	0.021	0.098	0.54	0.23	0.43	0.63	0.54	0.76	1.34
Lachnospiraceae FCS020 group	NO	NO	<0.001	0.002	0.84	0.00	0.00	0.01	0.01	0.04	0.06
Megasphaera	NO	YES	<0.001	0.006	0.79	0.00	0.00	1.31	0.00	0.02	0.99
Actinomyces	NO	NO	<0.001	0.009	0.75	0.00	0.01	0.04	0.01	0.04	0.13
Coprococcus 1	NO	YES	0.018	0.085	0.56	0.02	0.03	0.06	0.04	0.05	0.11
decreased											
Alistipes	YES	NO	<0.001	<0.001	-0.97	1.77	3.00	3.92	0.30	0.49	0.98
Odoribacter	YES	POTENTIAL	<0.001	0.002	-0.85	0.06	0.15	0.25	0.00	0.01	0.03
Parabacteroides	YES	POTENTIAL	<0.001	0.007	-0.77	0.39	0.93	1.62	0.05	0.16	0.24
Lachnospiraceae UCG-004	YES	YES	0.001	0.009	-0.74	0.40	0.60	1.76	0.05	0.16	0.39
Bacteroides	YES	NO	0.002	0.016	-0.70	7.76	14.17	20.39	2.08	4.66	9.92
Barnesiella	YES	YES	0.015	0.076	-0.57	0.47	1.07	2.95	0.04	0.20	0.75
Butyricimonas	NO	YES	0.010	0.054	-0.60	0.02	0.19	0.33	0.00	0.01	0.08
Sutterella	NO	NO	0.004	0.025	-0.66	0.00	0.20	0.41	0.00	0.00	0.07
Lachnospiraceae NC2004 group	NO	NO	0.003	0.022	-0.67	0.00	0.00	0.07	0.00	0.00	0.02
Lachnospiraceae UCG-008	NO	NO	0.001	0.011	-0.72	0.00	0.00	0.04	0.00	0.00	0.01
Clostridiales_unassigned	NO	POTENTIAL	<0.001	0.006	-0.78	0.01	0.02	0.04	0.00	0.00	0.00
Bacteria_unassigned	NO	n/a	<0.001	0.002	-0.86	0.02	0.03	0.08	0.00	0.00	0.00

Table S8 *Effect of inulin treatment on serum composition.*

	p	FDR	Cliff´s delta	A			B		
				Q1	median	Q3	Q1	median	Q3
asparagine	0.002	0.054	0.65	0.009	0.010	0.011	0.010	0.011	0.012
2-propanol	0.002	0.054	-0.65	0.043	0.046	0.050	0.041	0.043	0.047
butyric acid	0.005	0.061	0.62	0.161	0.344	0.535	0.322	0.440	0.827
propionic acid	0.006	0.061	0.62	0.399	0.730	1.288	0.841	1.148	2.481
glycerol	0.008	0.067	-0.58	0.061	0.072	0.101	0.054	0.072	0.086

Table S9 *Effect of inulin treatment on fecal metabolome: volatile organic compounds.*

	p-value	FDR	Cliff´s	A			B		
			delta	Q1	median	Q3	Q1	median	Q3
ethyl isobutyrate	0.004	0.264	0.65	0.024	0.051	0.128	0.067	0.121	0.216
propyl propionate	0.021	0.644	0.53	0.505	1.122	2.025	0.854	1.529	2.375
1-hexanol	0.034	0.691	-0.49	0.211	0.581	0.998	0.117	0.276	0.689

Only compounds meeting condition $AUC_{x,p}$ => 0.1% $AUC_{total,p}$ are shown. p, sample id.

Table S10 *Effect of inulin intervention on glucose metabolism in obese diabetic subjects*

	**pre-treatment**	**post-treatment**	**p-value**	**FDR**	**Cliffs´ delta**
Fasting glucose (mmol/l)	6.08 [0.86]	5.71 [0.9]	0.05	0.218	-0.43
2h OGTT glucose (mmol/l)	9.3 [2.8]	8.1 [3.7]	0.004	0.017	-0.65
AUC for OGTT glucose (mmol/l x 120min^{-1})	503 [136]	409 [255]	0.027	0.127	-0.49
AUC for OGTT insulin (mIU/l x 120min^{-1})	9321 [9926]	9088 [10053]	n.s.	N/A	0.20
Insulin (mIU/l)	16.7 [9.4]	14.8 [8.8]	n.s.	N/A	-0.16
C-peptide (pmol/l)	760 [388]	778 [333]	0.019	0.122	0.63
HbA1c (mmol/mol)	38 [7]	39 [5]	n.s.	N/A	0.34
Mcorr* (mg.kg^{-1}.min^{-1})	6.2 [2.4]	6.7 [4.0]	n.s.	N/A	0.17
MCR/I* (ml.kg^{-1}.min^{-1}. µU^{-1}.ml^{-1})	0.038 [0.025]	0.045 [0.028]	n.s.	N/A	0.23

Data are given as median [interquartile range]. AUC, area under the curve during oral glucose tolerance test; HbA1, glycated hemoglobin; Mcorr, glucose disposal space corrected and adjusted to fat free mass; MCR/I, metabolic clearance rate of glucose space corrected and adjusted to fat free mass divided by steady state insulinaemia; N/A, not applicable; n.s., not significant; *, values adjusted for fat-free mass.

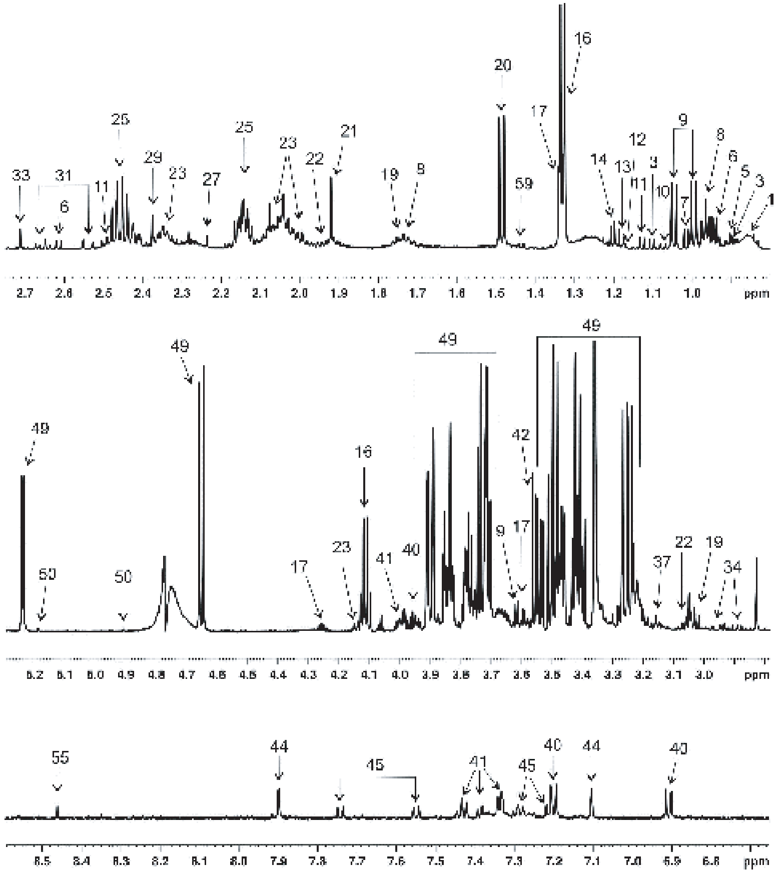

Supplementary Figure 1 *Representative ^1H NMR spectrum of serum with quantified metabolites*. Metabolite assignments for numbers are given in Table S1.

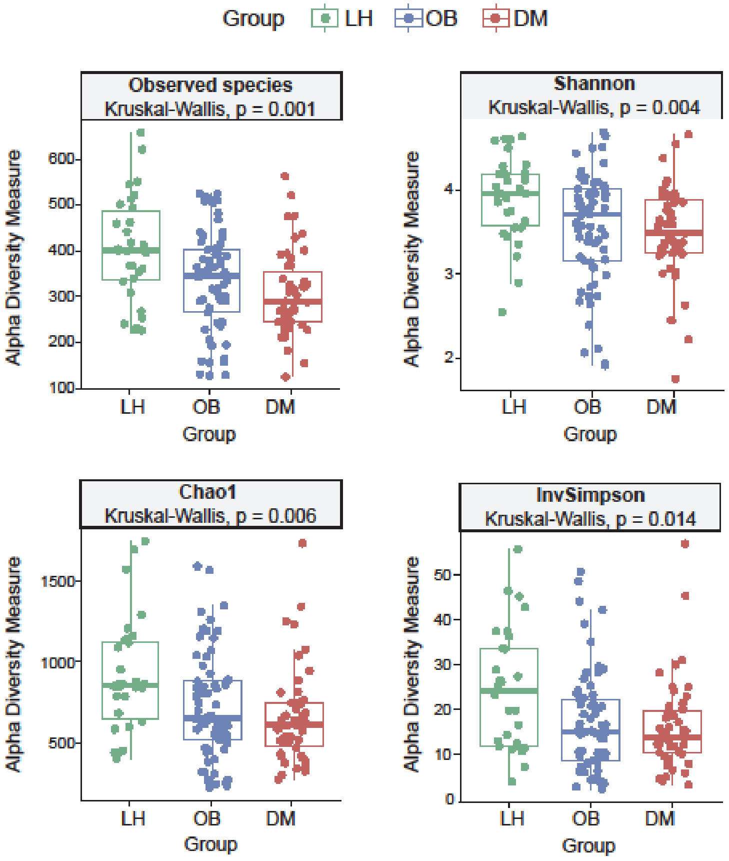

Comparisons	Dunn's multiple comparison test p-values			
	Observed species	Shannon	Chao1	InvSimpson
LH - OB	0.026	0.014	0.028	0.013
DM - LH	0.001	0.003	0.005	0.022
DM - OB	0.087	0.317	0.245	0.804

Supplementary Figure 2 *Alpha diversity of the gut microbiota*. The diversity indices were calculated on rarefied ASV data. The differences among groups were calculated using non-parametric ANOVA (Kruskal-Wallis test) followed by Dunn's multiple comparison test. ASV, amplicon sequence variant.

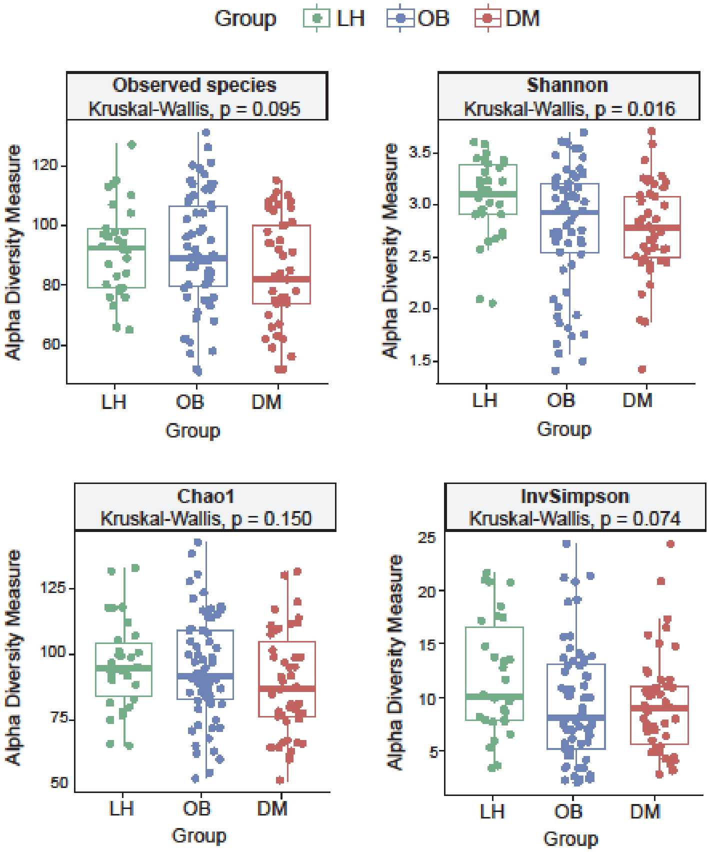

Comparisons	Dunn's multiple comparison test p-values			
	Observed species	Shannon	Chao1	InvSimpson
LH - OB	0.672	0.038	0.671	0.093
DM - LH	0.143	0.015	0.279	0.078
DM - OB	0.082	0.397	0.180	0.907

Supplementary Figure 3 *Alpha diversity of the gut microbiota*. The diversity indices were calculated on rarefied ASV data aggregated and classified to genus level. The differences among groups were calculated using non-parametric ANOVA (Kruskal-Wallis test) followed by Dunn test for testing pairwise differences. ASV, amplicon sequence variant.

A

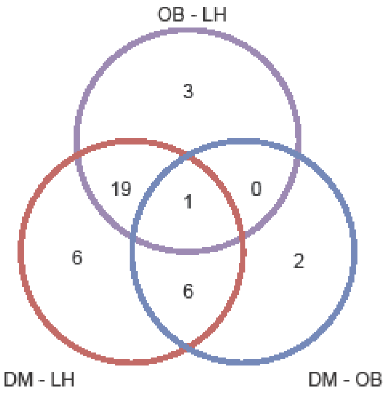

B

LH vs DM<br>LH vs OB<br>OB vs DM	LH vs DM<br>LH vs OB	DM vs LH<br>DM vs OB	OB - LH	DM - LH	OB vs DM
Blautia	*Anaerostipes*	*Christensenellaceae* R-7	*Dorea*	*Tyzzerella* 4	Family XIII AD3011 group
	Fusicatenibacter	*Lachnoclostridium*	*Marvinbryantia*	*Flavonifractor*	*Pseudobutyrivibrio*
	Lachnospiraceae_incertae sedis	Lachnospiraceae NK4A136	Lachnospiraceae_ unassigned	*Catenibacterium*	
	Lachnospiraceae FCS020	*Tyzzerella* 3		*Slackia*	
	Lachnospiraceae ND3007	*Megamonas*		*Mitsuokella*	
	Lachnospiraceae UCG-008	*Fusobacterium*		*Succinivibrio*	
	[*Eubacterium*] *hallii*				
	[*Ruminococcus*] *gauvreauii*				
	Faecalibacterium				
	Ruminococcaceae UCG-004				
	Erysipelotrichaceae UCG-003				
	Megasphaera				
	Desulfovibrio				
	Alloprevotella				
	Prevotella 7				
	Prevotellaceae_unassigned				
	Bacteroidales_unassigned				
	Bifidobacterium				
	Cyanobacteria_unassigned				

Supplementary Figure 4 *Bacteria specific/common for individual groups.* The uniqueness or overlap among groups is demonstrated using Venn diagram. Numbers within the circles show the number of bacteria fulfilling particular condition. Only bacteria selected by UDAA (FDR=>0.1) are shown.

A

Accuracy: **51.1 %**

	sensitivity	specificity
LH	66.0	79.5
OB	50.3	69.5
DM	42.6	75.6

B

Confusion matrix

	LH	OB	DM
LH	198	139	89
OB	54	322	181
DM	48	179	200

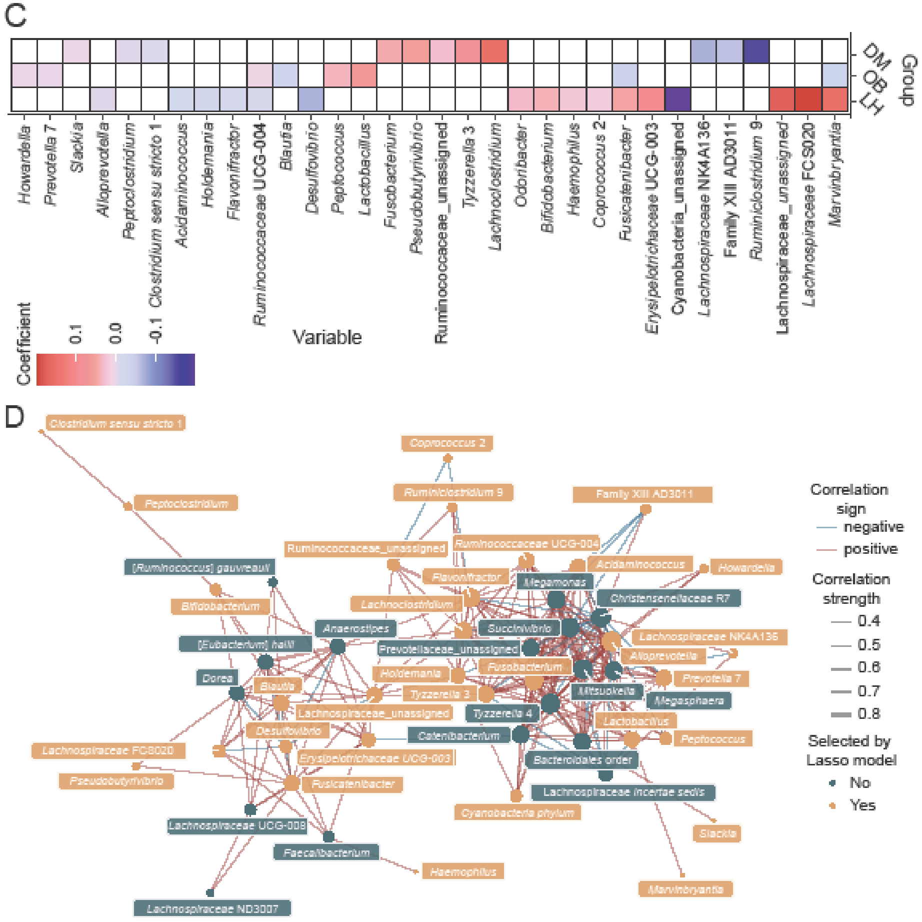

Supplementary Figure 5 *Classification into LH, OB and DM groups: Lasso model built on microbiome data (ASV aggregated to genus level).* A: Sensitivity and specificity metrics; B: Confusion matrix; C: Lasso coefficients; D: Correlation network based on Spearman correlation coefficients among significant variables selected by UDAA and Lasso model. Correlation analysis was performed on important variables from lasso model against significant variables from UDAA (adjusted p<=0.1) of the same dataset using correlation networks. |ρ| => 0.3 UDAA, univariate discrimination analysis.

A

Accuracy: **74.9 %**

group	sensitivity	specificity
LH	65.0	77.6
OB+DM	77.6	65.0

B

Confusion matrix

	LH	OB+DM
LH	195	249
OB+DM	105	861

C

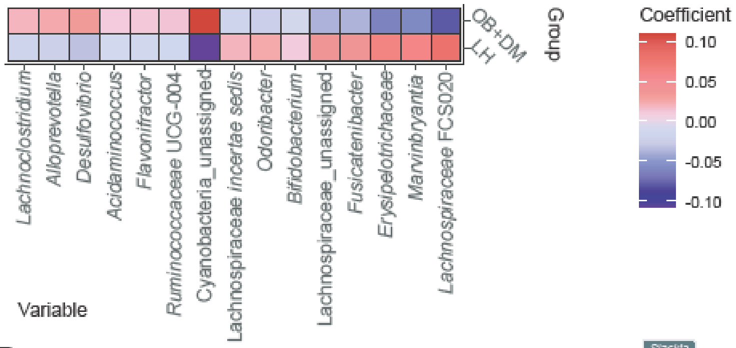

D

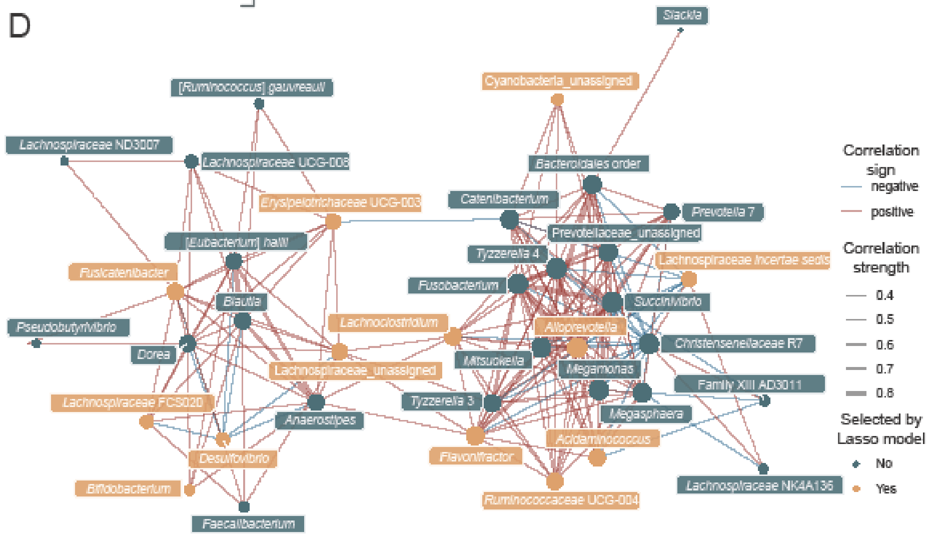

Supplementary Figure 6 *Classification into LH and (OB+DM) groups: Lasso model built on microbiome data (ASV aggregated to genus level.* A: Sensitivity and specificity metrics; B: Confusion matrix; C: Lasso coefficients; D: Correlation network based on Spearman correlation coefficients among significant variables selected by UDAA and Lasso model. Correlation analysis was performed on important variables from lasso model against significant variables from UDAA (adjusted p<=0.1) of the same dataset using correlation networks. |ρ| => 0.3 UDAA, univariate discrimination analysis.

A

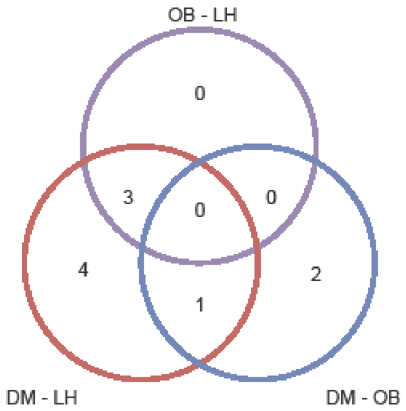

B

LH vs DM<br>LH vs OB<br>OB vs DM	LH vs DM<br>LH vs OB	LH vs DM<br>OB vs DM	LH vs OB	LH vs DM	OB vs DM
0	3	1	0	4	1
	Propyl acetate	Teradecanal		Humulene	
	Nonanoic acid			trans-Ocimene	Methyl pentanoate
	Decane			2-Octanol	
				Benzacetaldehyde	

Supplementary Figure 7 *VOCs specific/common for individual groups.* The uniqueness or overlap among groups is demonstrated using Venn diagram. Numbers within the circles show the number of VOCs fulfilling particular condition. Only VOCs selected by UDAA (FDR=>0.1) are shown.

A

Accuracy: **52.4 %**

	sensitivity	specificity
LH	48.0	88.2
OB	54.3	69.7
DM	52.8	68.0

B

Confusion matrix

	LH	OB	DM
LH	144	75	42
OB	67	326	142
DM	89	199	206

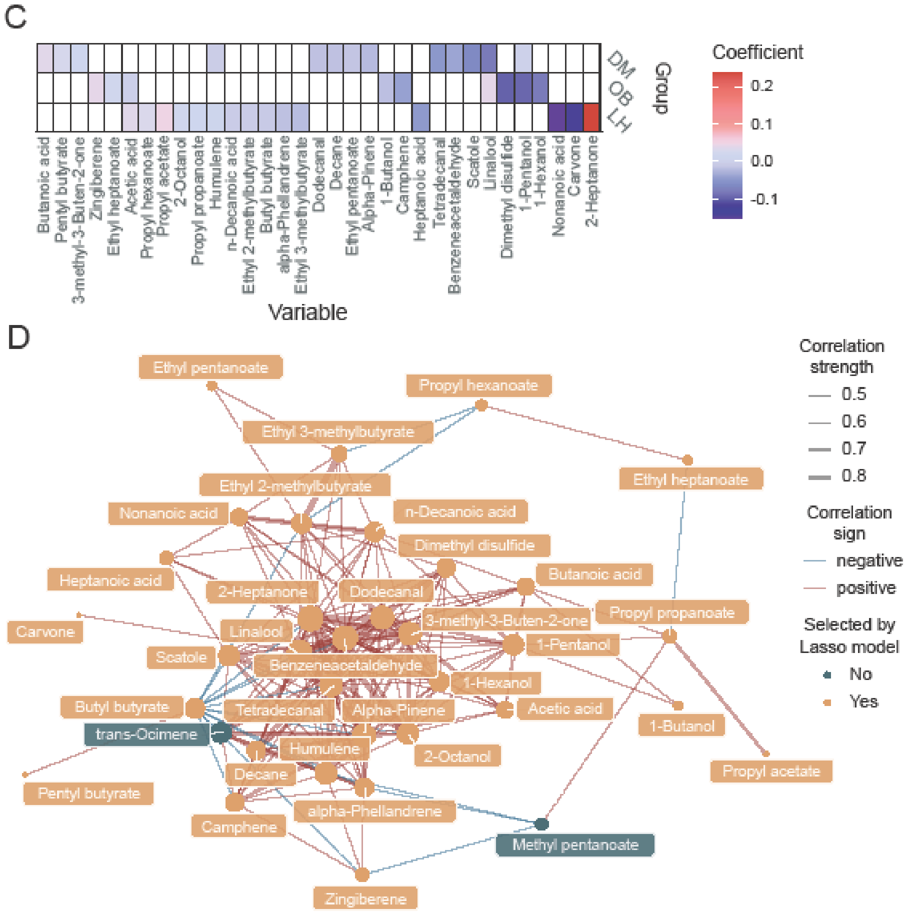

Supplementary Figure 8 *Classification into LH, OB and DM groups: Lasso model built on fecal metabolome data (VOCs).* A: Sensitivity and specificity metrics; B: Confusion matrix; C: Lasso coefficients; D: Correlation network based on Spearman correlation coefficients among significant variables selected by UDAA and Lasso model. Correlation analysis was performed on important variables from lasso model against significant variables from UDAA (adjusted p<=0.1) of the same dataset using correlation networks. |ρ| => 0.4 UDAA, univariate discrimination analysis.

A

Accuracy: **80.5 %**

group	sensitivity	specificity
LH	52.3	89.1
OB+DM	89.1	52.3

B

Confusion matrix

	LH	OB+DM
LH	157	108
OB+DM	143	882

C

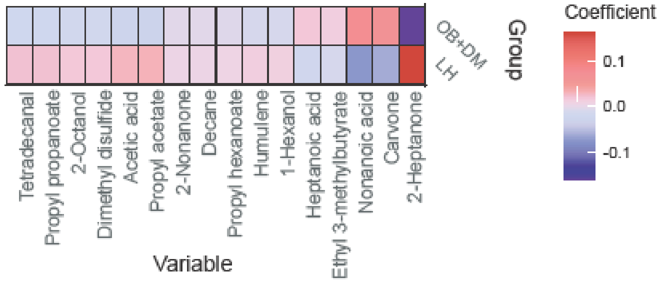

D

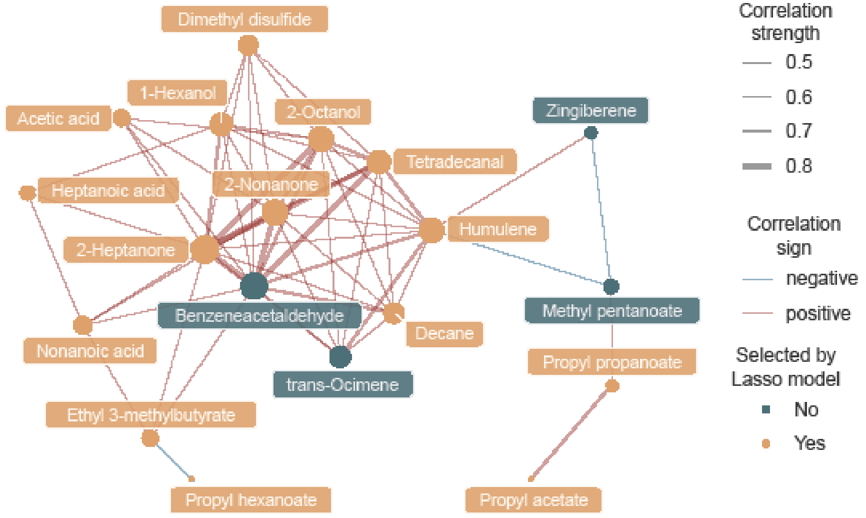

Supplementary Figure 9 *Classification into LH and (OB+DM) groups: Lasso model built on fecal metabolome data (VOCs).* A: Sensitivity and specificity metrics; B: Confusion matrix; C: Lasso coefficients; D: Correlation network based on Spearman correlation coefficients among significant variables selected by UDAA and Lasso model. Correlation analysis was performed on important variables from lasso model against significant variables from UDAA (adjusted p<=0.1) of the same dataset using correlation networks. |ρ| => 0.4 UDAA, univariate discrimination analysis.

A

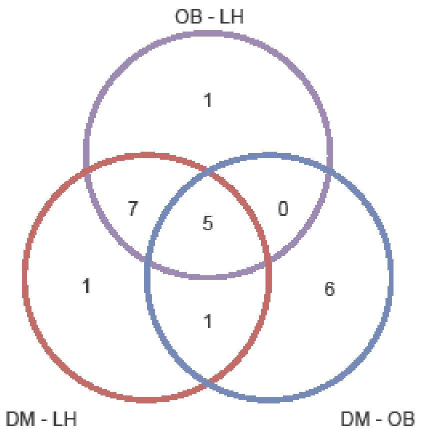

B

LH vs DM<br>LH vs OB<br>OB vs DM	LH vs DM<br>LH vs OB	LH vs DM<br>OB vs DM	LH vs OB	LH vs DM	OB vs DM
5	7	1	1	1	6
Glutamine	Succinic acid	Glycine	Ethanol	Pyruvate	Acetone
Mannose	Glycerol				2-Propanol
Lactate	Valeric acid				2-Oxoisovalerate
Alanine	Tyrosine				3-Methyl-2-oxovalerate
Glucose	Propionic acid				Formic acid
	Histidine				2-Hydroxybutyrate
	Asparagine				

Supplementary Figure 10 *Serum metabolites specific/common for individual groups.* The uniqueness or overlap among groups is demonstrated using Venn diagram. Numbers within the circles show the number of metabolites fulfilling particular condition. Only metabolites selected by UDAA (FDR=>0.1) are shown.

A

Accuracy: **73.8 %**

	sensitivity	specificity
LH	90.3	85.8
OB	71.7	78.4
DM	65.3	94.8

B

Confusion matrix

	LH	OB	DM
LH	280	134	22
OB	30	466	134
DM	0	50	294

C

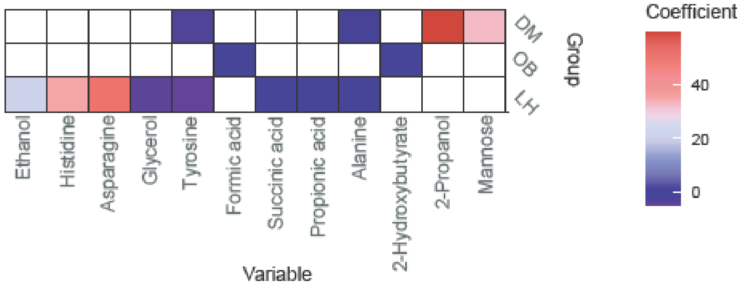

D

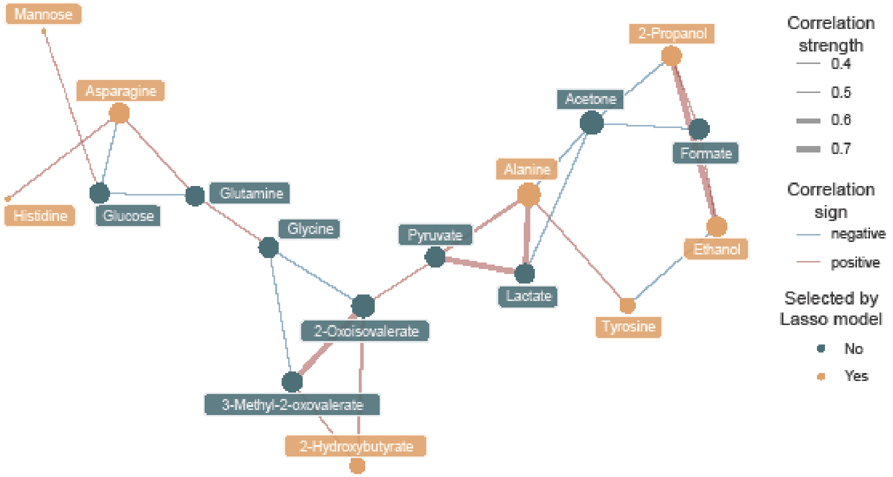

Supplementary Figure 11 *Classification into LH, OB and DM groups: Lasso model built on serum metabolome data.* A: Sensitivity and specificity metrics; B: Confusion matrix; C: Lasso coefficients; D: Correlation network based on Spearman correlation coefficients among significant variables selected by UDAA and Lasso model. Correlation analysis was performed on important variables from lasso model against significant variables from UDAA (adjusted p<=0.1) of the same dataset using correlation networks. |ρ| => 0.3 UDAA, univariate discrimination analysis.

A

Accuracy: **88.6 %**

group	sensitivity	specificity
LH	88.4	88.6
OB+DM	88.6	88.4

B

Confusion matrix

	LH	OB+DM
LH	274	125
OB+DM	36	975

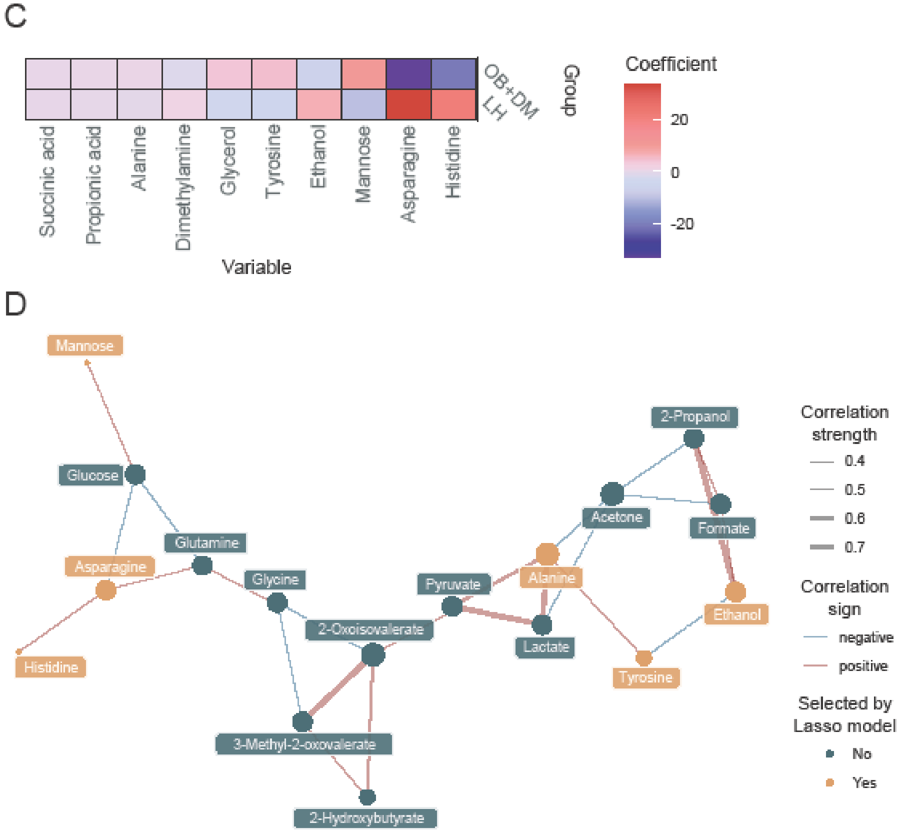

Supplementary Figure 12 *Classification into LH and (OB+DM) groups: Lasso model built on serum metabolome data.* A: Sensitivity and specificity metrics; B: Confusion matrix; C: Lasso coefficients; D: Correlation network based on Spearman correlation coefficients among significant variables selected by UDAA and Lasso model. Correlation analysis was performed on important variables from lasso model against significant variables from UDAA (adjusted p<=0.1) of the same dataset using correlation networks. |ρ| => 0.3 UDAA, univariate discrimination analysis.

A

Accuracy: **77.3 %**

	sensitivity	specificity
LH	88.2	89.4
OB	79.3	78.5
DM	65.7	95.3

B

Confusion matrix

	LH	OB	DM
LH	247	85	18
OB	31	476	109
DM	2	39	243

C

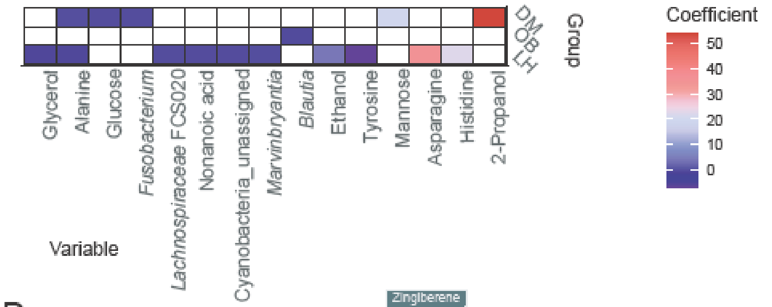

D

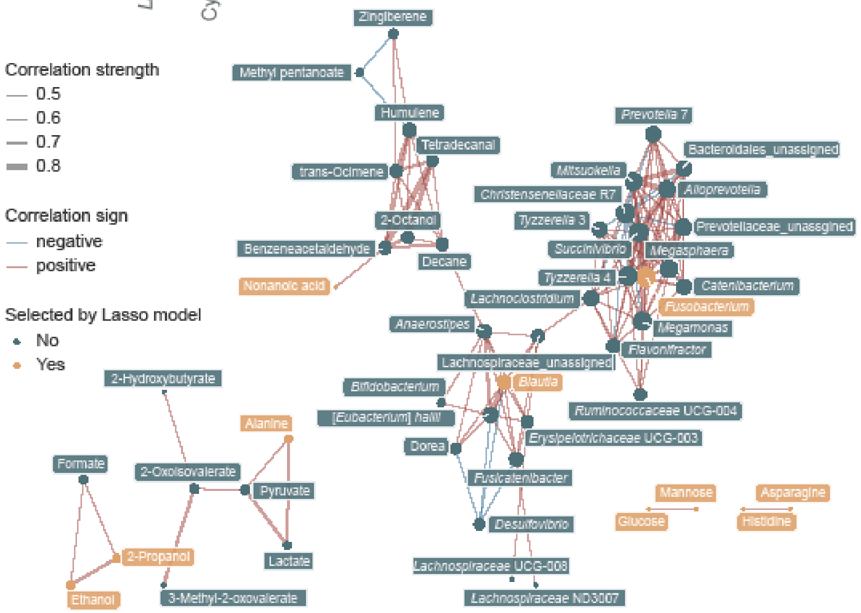

Supplementary Figure 13 *Classification into LH, OB and DM groups: Lasso model built on all variables.* A: Sensitivity and specificity metrics; B: Confusion matrix; C: Lasso coefficients; D: Correlation network based on Spearman correlation coefficients among significant variables selected by UDAA and Lasso model. Correlation analysis was performed on important variables from lasso model against significant variables from UDAA (adjusted p<=0.1) of the same dataset using correlation networks. |ρ| => 0.4 UDAA, univariate discrimination analysis.

A

Accuracy: **90.9 %**

group	sensitivity	specificity
LH	88.9	91.4
OB+DM	91.4	88.9

B

Confusion matrix

	LH	OB+DM
LH	249	83
OB+DM	31	887

C

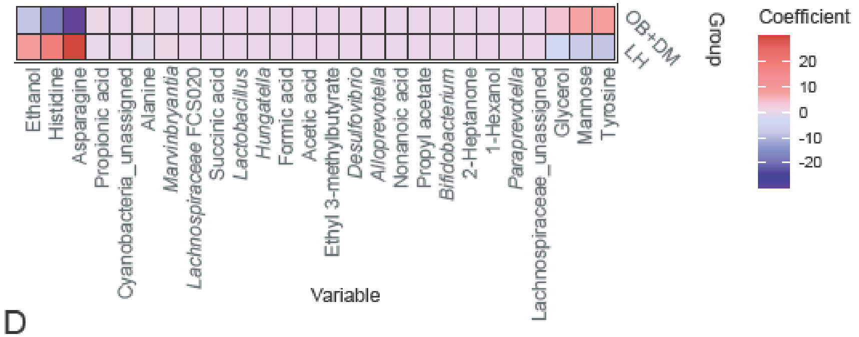

D

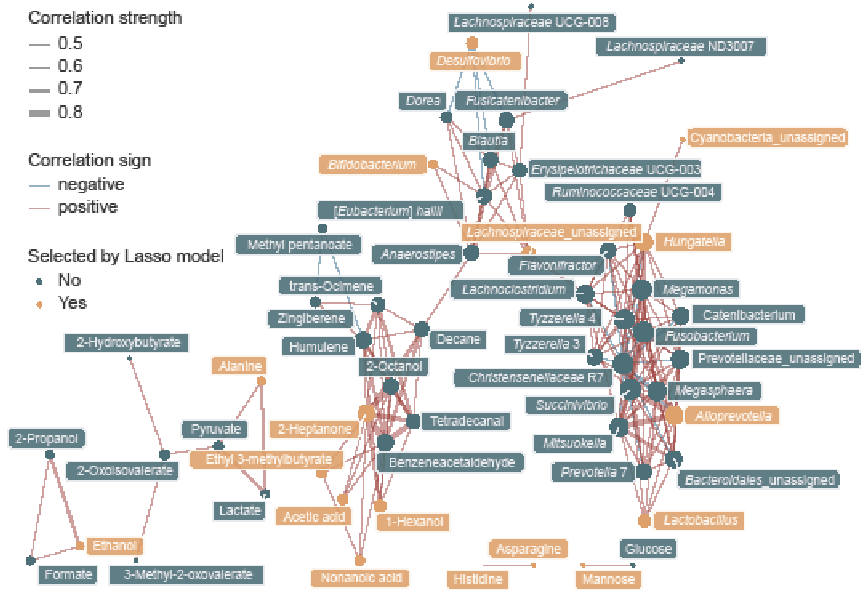

Supplementary Figure 14 *Classification into LH and (OB+DM) groups: Lasso model built on all variables.* A: Sensitivity and specificity metrics; B: Confusion matrix; C: Lasso coefficients; D: Correlation network based on Spearman correlation coefficients among significant variables selected by UDAA and Lasso model. Correlation analysis was performed on important variables from lasso model against significant variables from UDAA (adjusted p<=0.1) of the same dataset using correlation networks. |ρ| => 0.4 UDAA, univariate discrimination analysis.

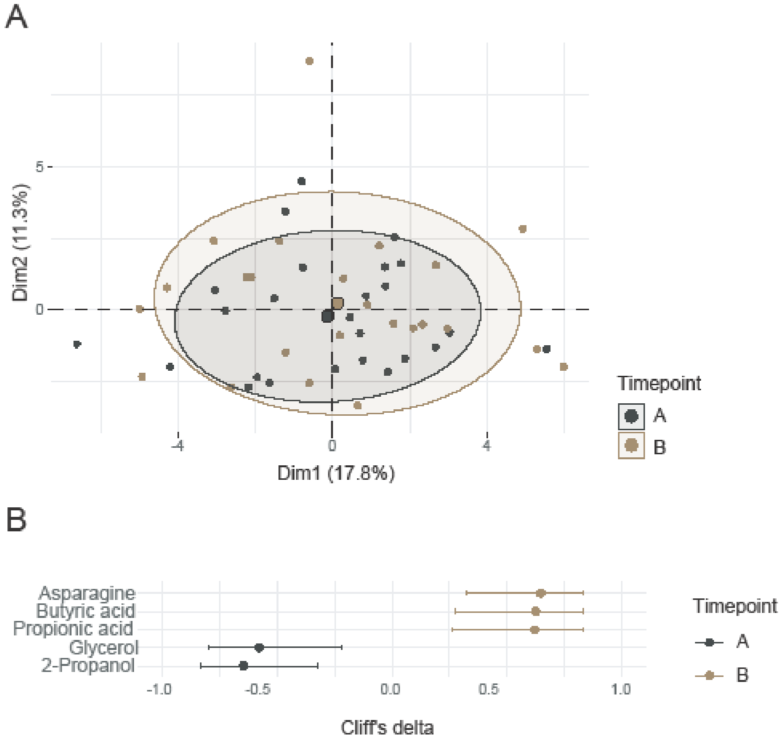

Supplementary Figure 15 *Effect of inulin on serum metabolome composition.* A: 2D PCA scores plot. The explained variance of each component is included in the axis labels. The large points represent the centroids of each group. B: Biomarker compounds. Biomarkers were generated from univariable discrimination analysis (FDR ≥ 0.1), with effect size estimated by Cliff's delta. A, time point prior to intervention; B, time point post-intervention.

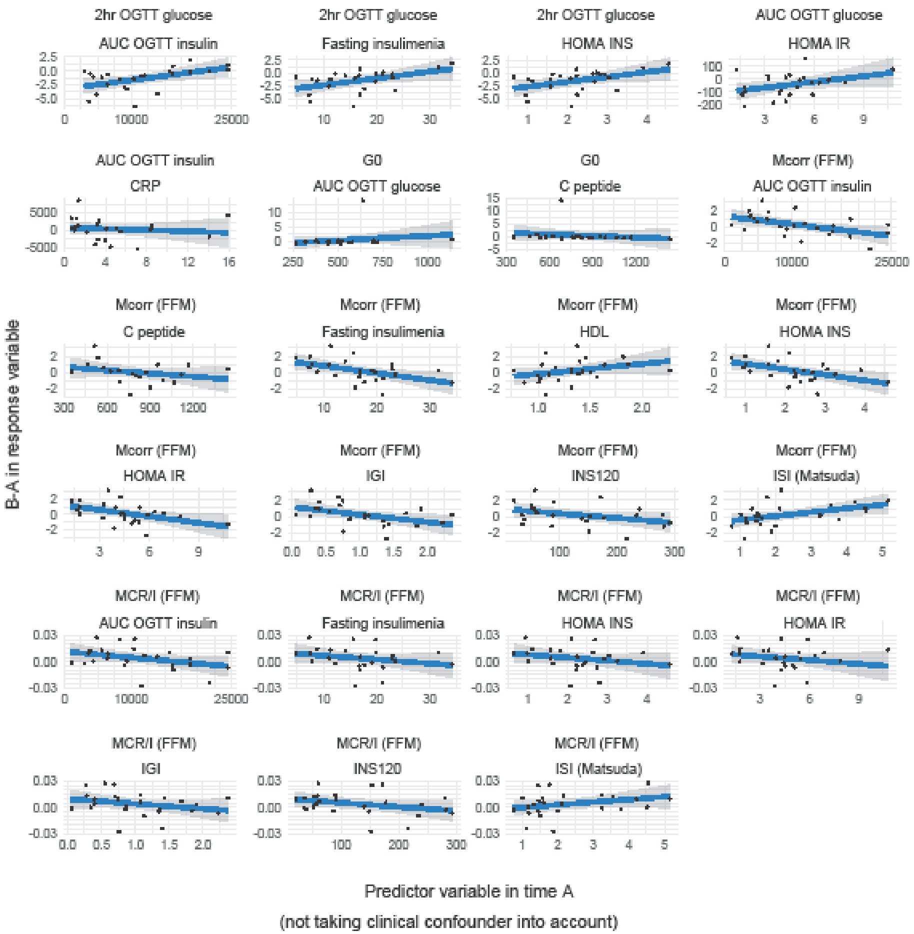

Supplementary Figure 16 *Predictive signatures for inulin intervention of glucose metabolism parameters and clinical variables*. Linear regression model of glucose metabolism parameters which were used as outcome variables and clinical parameters which were used as predictors. On the x axis there is predictor variable in time A (specific clinical parameter), on the y axis there is predicted difference of outcome variable (specific parameter of glucose metabolism) between its value in timepoint B and its value in timepoint A.

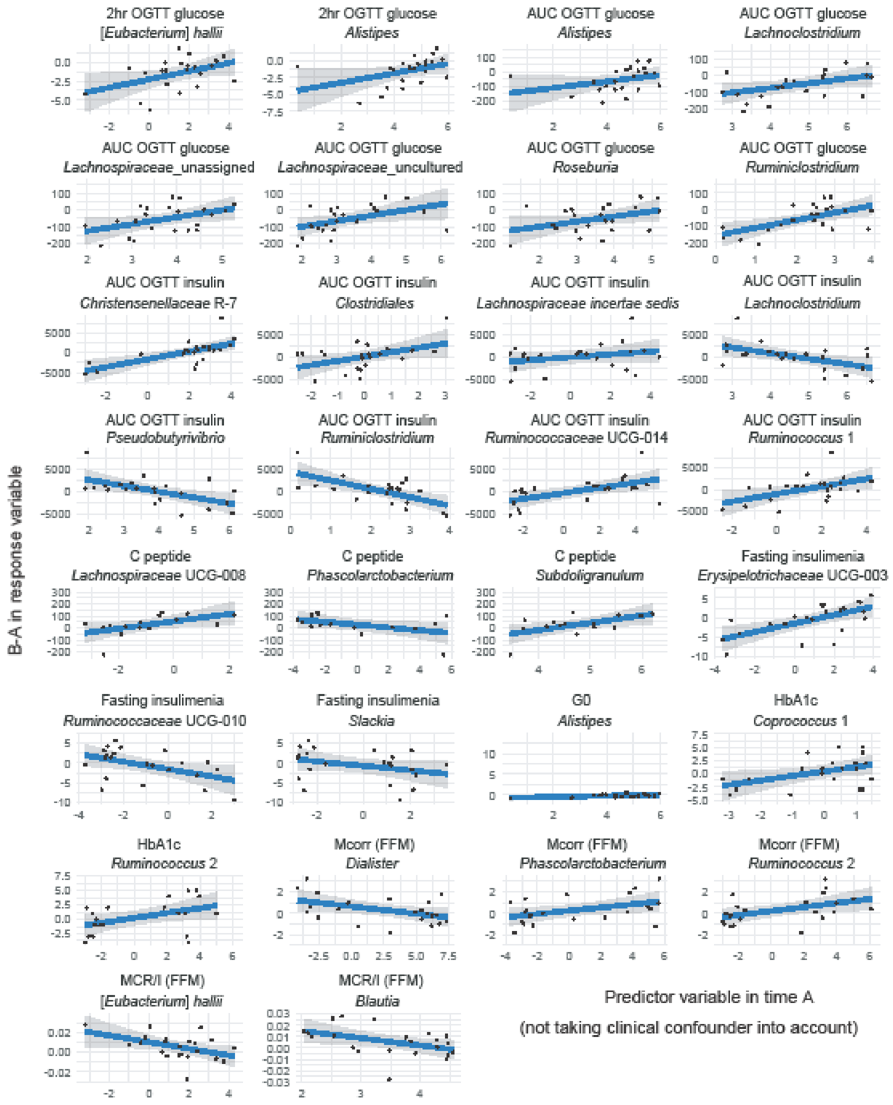

Supplementary Figure 17 *Predictive signatures for inulin intervention of glucose metabolism parameters and gut microbiota composition data*. Linear regression model of glucose metabolism parameters which were used as outcome variables and gut microbiota composition data (ASV aggregated to genus level) which were used as predictors. On the x axis there is predictor variable in time A (specific bacterial taxa), on the y axis there is predicted difference of outcome variable (specific parameter of glucose metabolism) between its value in timepoint B and its value in timepoint A.

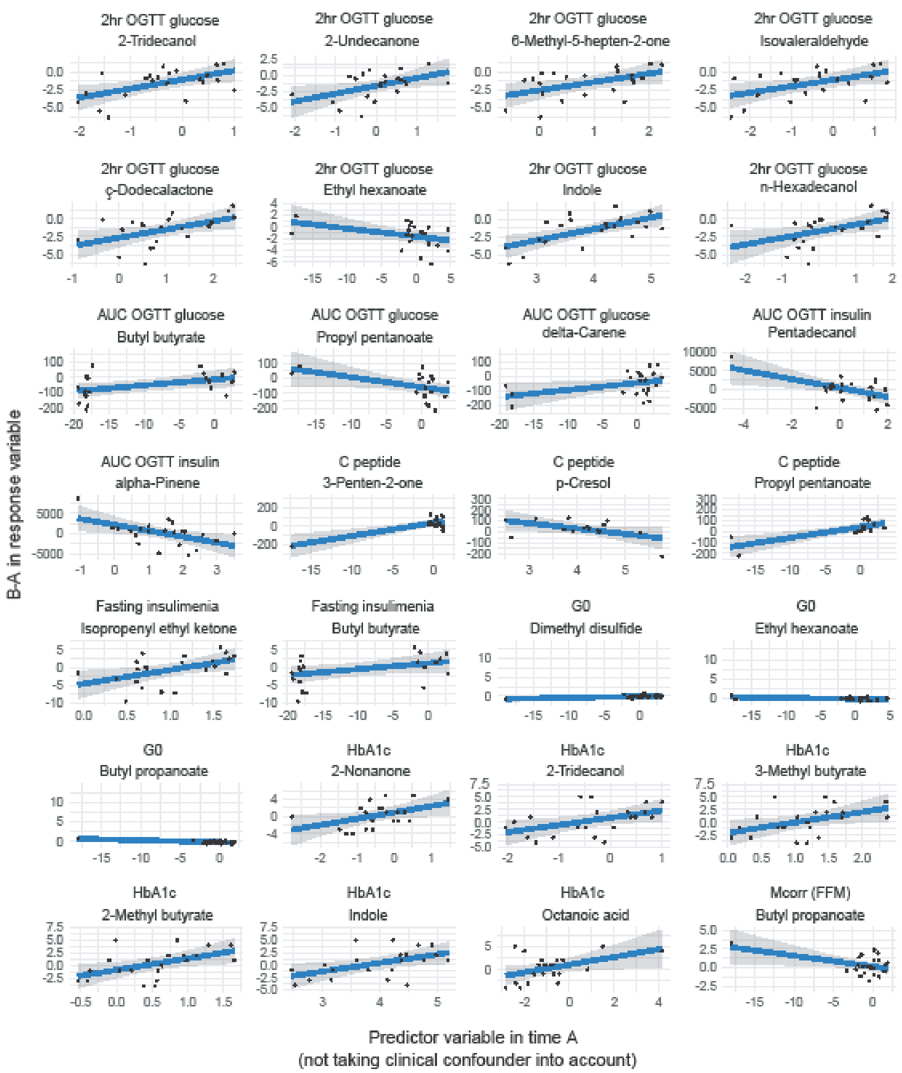

Supplementary Figure 18 *Predictive signatures for inulin intervention of glucose metabolism parameters and fecal metabolome data*. Linear regression model of glucose metabolism parameters which were used as outcome variables and fecal metabolome data (VOCs) which were used as predictors. On the x axis there is predictor variable in time A (specific fecal metabolite), on the y axis there is predicted difference of outcome variable (specific parameter of glucose metabolism) between its value in timepoint B and its value in timepoint A.

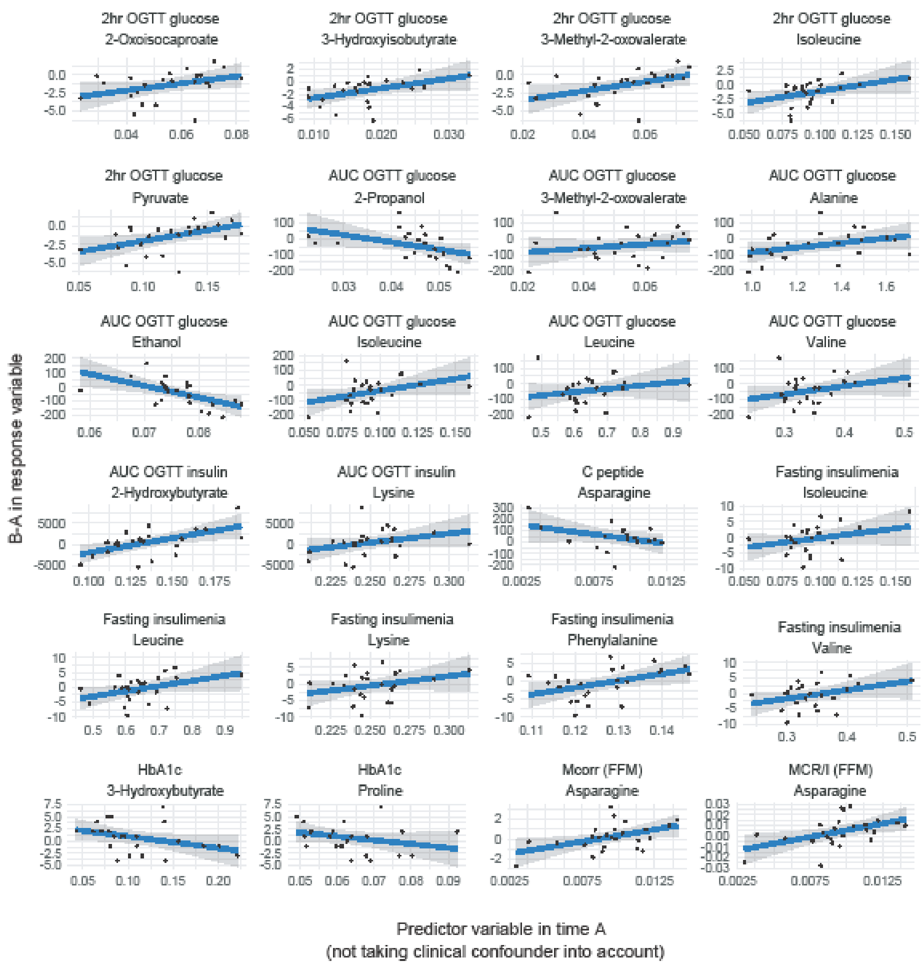

Supplementary Figure 19 *Predictive signatures for inulin intervention of glucose metabolism parameters and serum metabolome data*. Linear regression model of glucose metabolism parameters which were used as outcome variables and serum metabolome data which were used as predictors. On the x axis there is predictor variable in time A (specific serum metabolite), on the y axis there is predicted difference of outcome variable (specific parameter of glucose metabolism) between its value in timepoint B and its value in timepoint A.
